# Mitochondrial Dyes with Balanced Permeability-Fixability for Live, Fixed, and Expanded Multicellular Imaging

**DOI:** 10.1021/jacs.6c16881

**Published:** 2026-09-07

**Authors:** Jingting Chen, Yue Ma, Peng Chen, Biaoyi Yu, Ying Yu, Zhenzhen Zhang, Xiaomin Zhang, Buqing Ye, Jianzhong Jeff Xi, Xiaohong Peng, Zhixing Chen

**Affiliations:** † College of Future Technology, Institute of Molecular Medicine, National Biomedical Imaging Center, Beijing Key Laboratory of Cardiometabolic Molecular Medicine, 12465 Peking University, Beijing 100871, China; ‡ Peking-Tsinghua Center for Life Sciences, Academy for Advanced Interdisciplinary Studies, Peking University, Beijing 100871, China; § Genvivo Biotech (PuHaiJingShan), Nanjing 211800, China; ∥ School of Basic Medical Sciences, 481870 Shenzhen University Medical School, Shenzhen 518060, China; ⊥ State Key Laboratory of Natural and Biomimetic Drugs, Institute of Molecular Medicine, Department of Biomedical Engineering, Peking University, Beijing 100871, China; # Key Laboratory of Brain, Cognition and Education Sciences, Ministry of Education, China; Institute for Brain Research and Rehabilitation, and Guangdong Key Laboratory of Mental Health and Cognitive Science, 12451 South China Normal University, Guangzhou 510631, China; & State Key Laboratory of Membrane Biology, Peking University, Beijing 100871, China

## Abstract

Mitochondrial inner-membrane ultrastructure plays a central role in cellular metabolism, aging, and cell death. However, super-resolution imaging of mitochondria after fixation remains challenging: commercially available fixable probes often lack sufficient reactivity for strong retention during fixation, whereas state-of-the-art probe PKMO FX, despite high fixation efficiency, suffers from low cellular permeability due to the introduction of highly hydrophilic groups. Here, we overcome this “permeability-fixability trade-off” by introducing a rational design strategy centered on amide-to-ester substitution. We report mitochondrial probes, PK Mito 590 FIX and PK Mito 647 FIX, which leverage optimized lipophilic ester linkages to achieve rapid mitochondrial labeling kinetics (labeling within 10 min) and superior aldehyde cross-linking efficiency (>90% signal retention). This molecular engineering enables a seamless transition from live-cell dynamics to post-fixation super-resolution microscopy with unprecedented signal-to-background ratios. Crucially, the enhanced permeability of these probes unlocks post-fixation imaging of multicellular samples, allowing the visualization of mitochondrial architectures within patient-derived cell clusters and isolated mouse islets. Furthermore, we demonstrate that these probes withstand the harsh polymerization conditions of expansion microscopy (ExM), democratizing mitochondrial ultrastructural imaging via standard confocal platforms. Collectively, this toolkit bridges the gap between physiological dynamics and structural definition, offering a versatile platform for multiscale interrogation of mitochondrial biology in both adherent cells and complex multicellular systems.

## Graphical abstract

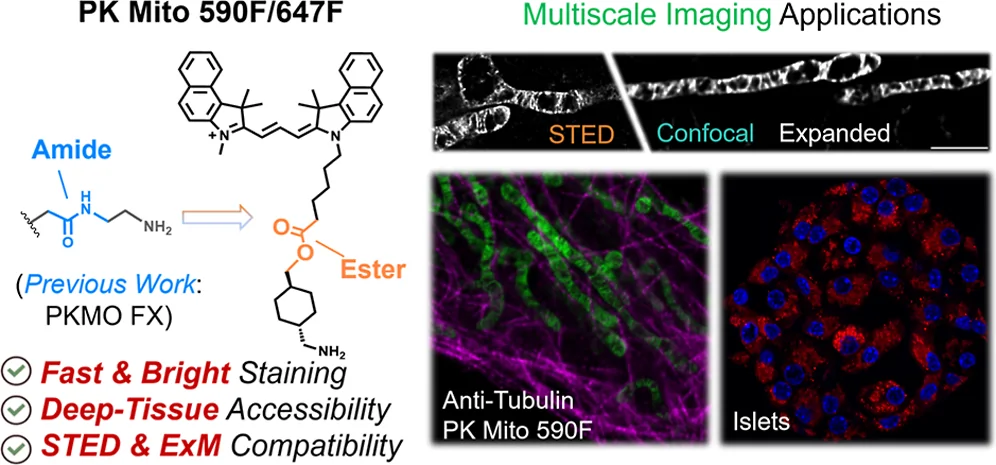

## Introduction

The physiological state of mitochondria is intrinsically linked to their intricate inner membrane architectures,^1^ particularly the cristae, which undergo dynamic remodeling in response to metabolic demands, stress, and disease.^2–4^ The advent of super-resolution microscopy (SRM),^5^ including stimulated emission depletion (STED) nanoscopy,^6,7^ has revolutionized our ability to resolve these subdiffraction structures.^8,9^ A complete understanding of mitochondrial biology demands not only single snapshots but also the spatiotemporal correlation of rapid physiological events with precise ultrastructural analysis. Live imaging of mitochondria followed by chemical fixation is an indispensable strategy for correlating dynamic cellular events with static structural analysis, as it allows for the preservation of physiological states while enabling downstream high-resolution or multimodal imaging.

Despite its conceptual elegance, this “live-to-fixed” paradigm is limited by a key chemical challenge: the scarcity of fluorescent probes that are simultaneously bioavailable and chemically fixable. Conventional mitochondrial dyes such as MitoTrackers,^10^ despite containing fixable benzyl chloride moieties, still suffer from substantial signal loss or leaching upon chemical fixation, rendering them unsuitable for post-fixation super-resolution imaging applications. Previous studies developed Mito-TEM and Mito-TEM 2.0 by introducing reactive aldehyde groups for mitochondrial anchoring, providing relevant precedents for improving probe retention.^11,12^ These studies highlight the value of chemical anchoring for maintaining stable mitochondrial labeling during prolonged imaging. Our group previously introduced PKMO FX, a prototypical probe capable of surviving fixation for STED imaging.^13^ While effective in principle due to amine-aldehyde cross-linking chemistry,^14^ PKMO FX incorporated hydrophilic amines to ensure fixability, which inadvertently compromised its membrane permeability. This created a restrictive trade-off: the probe functioned well in readily transfectable monolayers but failed to penetrate the complex, dense biological barriers characterizing physiologically relevant models, such as cell clusters, thick organoids, or tissues.

Drawing inspiration from medicinal chemistry strategies used to optimize the cellular uptake of PROTACs and peptide drugs,^15,16^ we hypothesized that replacing the hydrophilic amide linkages—standard in dye conjugation—with lipophilic ester bonds would fundamentally alter the probe’s physicochemical behavior. We posited that this “amide-to-ester” substitution strategy would accelerate passive diffusion across the lipid bilayer for rapid staining, while maintaining, or even enhancing, the availability of amine groups for efficient aldehyde cross-linking during fixation.

Herein, we present PK Mito 590 FIX and PK Mito 647 FIX, a new palette of bioavailable and fixable mitochondrial probes designed through an amide-to-ester substitution strategy combined with optimization of the amine hydrophobicity. These probes exhibit orders-of-magnitude improvements in mitochondrial labeling kinetics and signal retention compared to their amide-based counterparts. This enhanced bioavailability not only facilitates high-quality STED imaging in diverse cell lines but also unlocks the direct visualization of mitochondria in tissue contexts, including patient-derived tumor cell clusters and isolated mouse islets. Furthermore, we demonstrate the chemical robustness of these probes by applying them to expansion microscopy (ExM). By surviving the radical polymerization and proteolysis steps of ExM, PK Mito FIX dyes enable the resolution of mitochondrial ultrastructure using standard diffraction-limited confocal microscopes.

## Results

### Design and Optimization of the Fixable Mitochondrial Dyes

In our prior work, we have demonstrated that Cy3.5 is a privileged fluorophore for mitochondrial dye^17^ and its primary amine derivative PKMO FX enables the fixation-driven cross-linking with aldehydes.^13^ However, the introduction of highly hydrophilic amino groups reduces the cellular permeability of PKMO FX. We sought to further deliver a palette of fixable mitochondrial probes with improved cell permeability and cross-linking efficiency. We hypothesized that, based on the principles governing passive diffusion across lipid bilayers,^18,19^ molecules with higher lipophilicity would not only enter cells more rapidly but also diffuse out of mitochondria more slowly following the addition of fixative. Such hydrophobic tuning would enhance their cross-linking with the aldehyde fixative, ultimately boosting the post-fixation imaging performance (Figure 1A). To this end, we are inspired by the molecular designs in medicinal chemistry,^20,21^ where amide linkages are oftentimes too hydrophilic to be permeable. To avoid the desolvation penalties of amide bonds when passing the lipid bilayer, ester linkages have emerged as a strategy to optimize the permeability and cellular activity of PROTACs and peptide drugs.^15,16^ Therefore, we plan to screen primary amine compounds with different lipophilicities, as well as the coupling bonds between amines and fluorophores.

**Figure 1:**
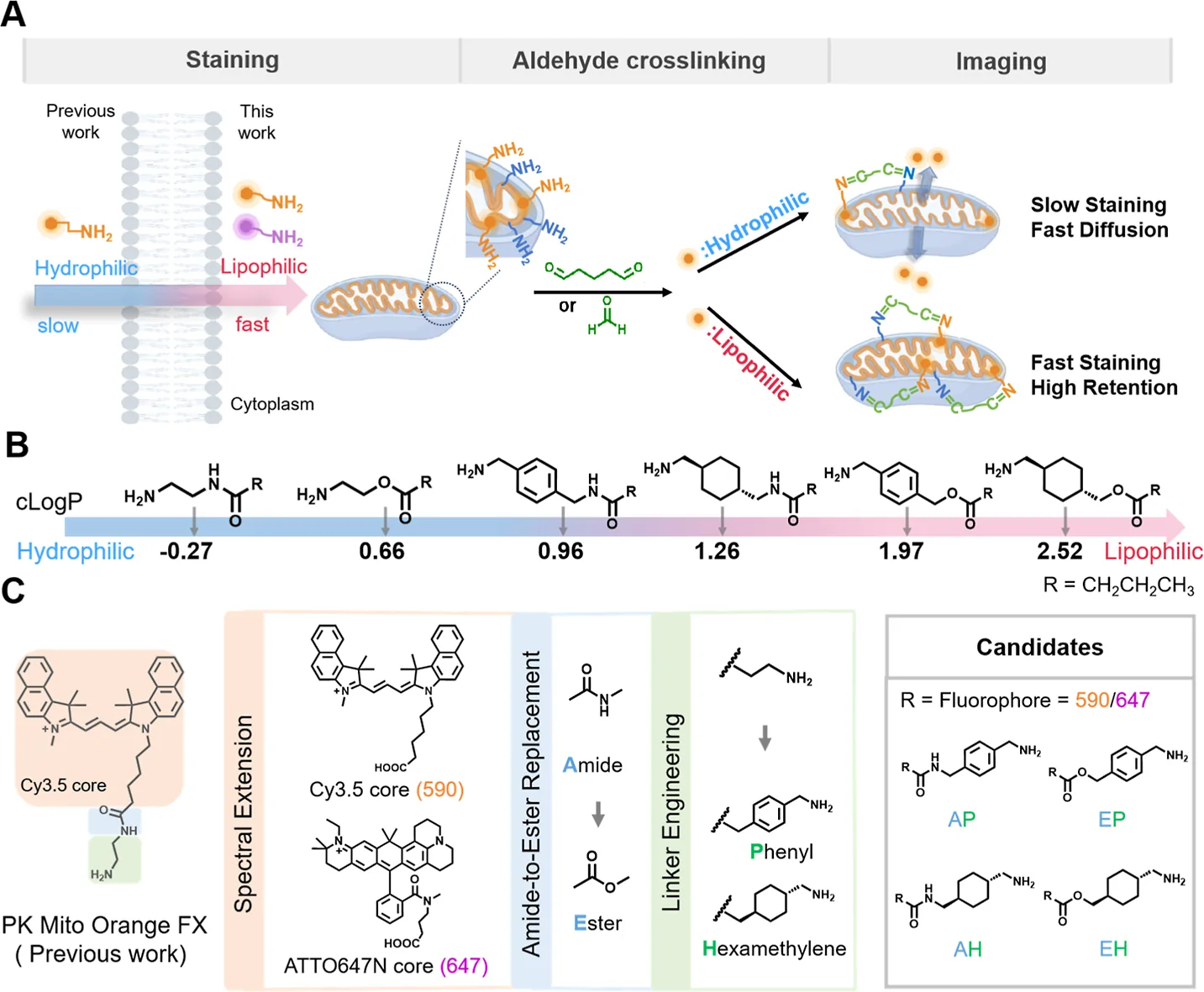
Design of fixable mitochondrial palette via spectral expansion and linker engineering. (A) Fixation-driven cross-linking is a privileged strategy for high-density staining of mitochondria, where the deliberately tuned lipophilicity is a key parameter for fast staining and high retention during fixation. (B) Assessing the lipophilicity of various linkers using cLogP analysis in ChemDraw. (C) Structural optimization strategies give rise to candidates combining different fluorophores (Cy3.5, ATTO647N), amide-to-ester replacement (amide bond, ester bond), and linker engineering (phenyl, hexamethylene).

We first calculated the lipid–water partition coefficients of different primary amines conjugated with propyl groups via amide or ester bonds to give cLogP as a parameter (Figure 1B), where a higher cLogP value indicates greater lipophilicity of the molecule. The results showed that amines with cyclohexane or benzene as spacers are more lipophilic than the ethylenediamine spacer (cLogP: 0.96, 0.66 vs −0.27), and the cLogP values further increase when they are conjugated via ester bonds (cLogP 2.52 vs 1.26, 1.97 vs 0.96). Guided by this prediction, we selected two fluorophores that have been shown to be privileged in mitochondrial super-resolution imaging, Cy3.5 and ATTO647N,^17,22^ and designed a panel of their amine derivatives featuring different linkers (Figure 1C). These molecules can be chemically synthesized via modular coupling reactions. For simplicity, we use the following abbreviations to refer to different dyes: 590 represents Cy3.5 fluorophore, and 647 represents ATTO647N fluorophore. A for amide linkage, E for ester linkage, P for amines with benzene as the spacer, and H for amines with cyclohexane as the spacer (Figure S1).

### Linker Engineering Generally Boosts Cell Permeability and Post-fixation Retention of Mitochondrial Probes

First, we systematically assessed the permeability of our new dye library. Each mitochondrial dye was incubated with live HeLa cells at the same concentration (300 nM) for different durations (1–20 min). After washing off the dyes with PBS, confocal imaging was performed under identical imaging parameters. The images were quantified by comparing mitochondrial brightness at different staining time points. The four ester-linked dyes (590-EP, 590-EH, 647-EP, and 647-EH) exhibit significantly higher mitochondrial signals than the five amide-linked dyes (PKMO FX, 590-AP, 590-AH, 647-AP, and 647-AH) at every time point (Figures 2A,B, S2 and S3). These results indicate that ester linkages can greatly boost the bioavailability of the mitochondrial dyes compared to their amide counterparts. Specifically, 590-EP showed 12× higher brightness than 590-AP after 10 min of staining, while 590-EH showed 17× higher brightness than 590-AH. The labeling brightness of 590-EH was slightly higher than that of 590-EP. Notably, the enhancement effect of amide-to-ester substitution on cellular permeability was more pronounced when applied to the ATTO647N fluorophore: after 20 min of staining, 647-EP showed 17× higher brightness than 647-AP, while 647-EH exhibited 29× higher brightness than 647-AH. The labeling brightness of 647-EP was higher than that of 647-EH at each time point, which was contrary to the results of the Cy3.5-based fluorophore. Specifically, after 5 min of short-term staining, the ester-linked dyes provided a high signal-to-noise ratio (SNR) for mitochondria, whereas the amide-linked dyes failed to visualize mitochondria without additional SNR enhancement (Figures 2C,D, S2 and S3). Ester-linked dyes, especially 590-EH and 647-EP, represent a substantial improvement over previous protocols that required verapamil supplementation and high-concentration staining for extended periods, demonstrating a markedly simplified and more efficient labeling approach for live-cell mitochondrial imaging, which are promising candidates for the next generation of fixable mitochondrial dyes.

**Figure 2:**
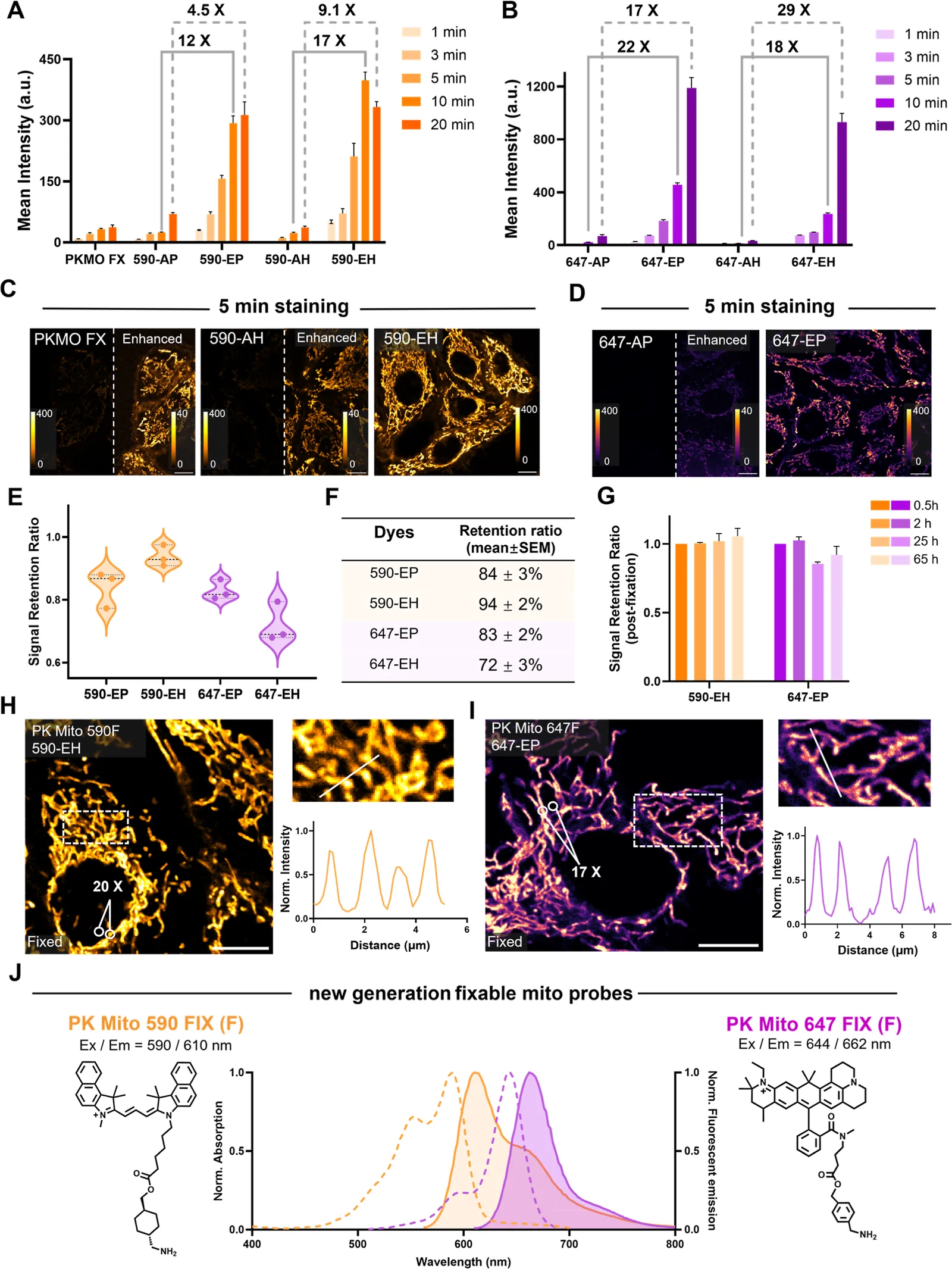
Lipophilic ester linkers render mitochondrial dyes more permeable in live cells and better retained during fixation. (A,B) Mitochondrial fluorescence intensity quantification for Cy3.5-based (A) or ATTO647N-based (B) dye candidates after different staining durations (1–20 min) in live HeLa cells. Staining was performed at 300 nM, followed by PBS wash before imaging. Fold changes indicate signal enhancement of amide-to-ester substitution after 10 or 20 min staining, as indicated. Data are presented as mean ± SEM. (C,D) Confocal imaging of live HeLa cells stained with Cy3.5-based (C) or ATTO647N-based (D) dye candidates for 5 min. Enhanced and same brightness and contrast images are shown. (Scale bars, 10 μm.) (E) Violin plots show signal retention ratios after fixation of HeLa cells stained with 590/647-EP/EH candidates. (F) Quantitative retention ratios (mean ± SEM) for each dye candidate. (G) Time-course analysis of signal retention ratio after fixation. (mean ± SEM). (H, I) HeLa cells stained with 590-EH (also PK Mito 590 FIX) (H) or 647-EP (also PK Mito 647 FIX) (I). Circled regions and line profiles along the indicated white lines highlight high signal-to-background ratios. (Scale bars, 10 μm.) (J) Structure and normalized absorption and fluorescence spectra of PK Mito 590 FIX (Ex = 590 nm, Em = 610 nm) and 647 FIX (Ex = 644 nm, Em = 662 nm).

Furthermore, to rigorously evaluate the mitochondrial labeling specificity of the four ester-linked dyes, colocalization studies were conducted in HeLa cells using Mito Tracker Red (MTR) or Mito Tracker Deep Red (MTDR) as reference mitochondrial markers. As shown in Figures S4 and S5, all the ester-linked probes showed high degrees of colocalization with Mitotracker-labeled mitochondria, with Pearson’s correlation coefficients (PCC) values greater than 0.94, which indicates that all ester-linked probes can effectively and specifically label mitochondria in live cells.

After observing the superiority of ester-linked mitochondrial dyes in live-cell staining, we further evaluated the signal retention capability of four ester-linked dyes following the addition of glutaraldehyde (GA) fixative. HeLa cells stained with 590-EP, 590-EH, 647-EP or 647-EH were subjected to GA fixation, respectively. Confocal imaging of the same cells both before and after fixation revealed that all four amine-conjugated mitochondrial probes exhibited excellent signal retention after fixation (Figures S6 and S7). The SNR of their post-fixation images is comparable to that of live-cell imaging, and significantly superior to that of MTR or MTDR, classic fixable dyes. Further statistical analysis of the mitochondrial fluorescent signal retention rate showed that 590-EH and 647-EP outperformed in the series, retaining 94% and 83% respectively of their initial fluorescence intensity after GA fixation (Figure 2E,F). Normalized fluorescence intensity distributions along the line in fixed cells further indicated that 590-EH and 647-EP exhibited superior signal-to-background ratios. The signal-to-background ratios were as high as 20× and 17× (Figure 2H,I), respectively, both significantly higher than the 8× of the previous fixable dye PKMO FX. Overall, 590-EH and 647-EP demonstrated the best live-cell permeability, mitochondrial fluorescence retention, and signal-to-background ratios upon fixation. Consequently, 590-EH (Ex = 590 nm, Em = 610 nm) and 647-EP (Ex = 644 nm, Em = 662 nm) were designated as PK Mito 590 FIX and PK Mito 647 FIX, respectively (Figure 2J).

To understand the molecular basis underlying their fixation performance, we next investigated the chemical properties governing probe retention during the fixation process. A major challenge for mitochondrial fixation is that the loss of membrane potential causes rapid dye efflux before cross-linking can occur. We hypothesized that increased probe lipophilicity would slow down this molecular diffusion out of depolarized mitochondria, thereby providing sufficient time for covalent cross-linking with the fixative. To test this, cells labeled with PK Mito 590 FIX or PKMO FX were treated with 20 μM FCCP to induce rapid depolarization. PK Mito 590 FIX showed a significantly slower fluorescence decrease (Figure S8), confirming that its higher lipophilicity retards dye efflux, suggesting superior fixation performance for PK Mito 590 FIX.

To verify that signal retention was not merely an artifact of physical entrapment, GA-fixed cells were treated with 5% Triton X-100 to comprehensively solubilize the lipid bilayers. While noncovalent trapping would result in complete signal loss under these harsh conditions, quantitative analysis showed that PK Mito 590 FIX and PK Mito 647 FIX retained ∼72% and ∼86% of their fluorescence, respectively. Furthermore, mitochondrial morphology remained well-preserved (Figure S9), providing definitive evidence of robust covalent cross-linking to the protein matrix.

Given the inherent hydrolytic susceptibility of esters, we systematically evaluated the stability of these ester-linked mitochondrial dyes in live cells, fixed cells, and aqueous solutions (Figures S10–S13). During a 24 h live-cell incubation, PK Mito 590F and PK Mito 647F maintained strictly specific mitochondrial targeting without diffuse cytosolic background, ruling out undesired ester hydrolysis. Most importantly, STED nanoscopy performed on both live and GA-fixed cells 24 h post-staining successfully resolved fine mitochondrial ultrastructures, demonstrating the stability of the ester linkage during prolonged incubation (Figure S10). Had ester hydrolysis occurred, loss of the conjugated primary amine moiety would have abolished the fixation capability of the probes. These results confirm the chemical and photophysical stability of the fluorophore-ester conjugates under prolonged physiological conditions. Furthermore, longitudinal imaging of fixed cells labeled with 590-EH and 647-EP revealed that from 0.5 to 65 h post-fixation (Figures S11, S12), the fluorescence of 590-EH remained unchanged, while 647-EP retained over 92% of its initial intensity (Figure 2G). Finally, LC–MS analysis confirmed that 150 μM aqueous solutions of both probes remained stable for over 4 days without detectable hydrolysis (Figure S13).

### PK Mito 590 FIX and 647 FIX Enable STED Imaging of Mitochondrial Ultrastructure in Fixed Cells

With the high labeling density rendered by the enhanced permeability, PK Mito 590 FIX and PK Mito 647 FIX were tested for super-resolution STED imaging of the mitochondrial inner membrane. First, HeLa cells were stained with PK Mito 647 FIX (300 nM) for 15 min, followed by a single PBS wash and fixation with 2.5% GA. Using STED microscopy, we successfully captured high-contrast images of brightly labeled mitochondrial cristae in fixed HeLa cells (Figure 3A). In parallel, PK Mito 590 FIX also exhibited excellent performance in STED imaging of mitochondrial cristae in fixed cells, enabling unambiguous visualization of cristae architectures. Beyond single-shot STED imaging, we performed z-stack STED imaging of PK Mito 590/647 FIX-labeled cells to acquire extended *z*-direction image stacks of the whole cells. By acquiring 10 individual optical sections at a step size of 200 nm, these z-stack recordings revealed a highly ordered organization of mitochondrial cristae network within cells (Figures 3B, S14,S15). Driven by the stringent photostability requirements of such continuous acquisitions, we further benchmarked their photobleaching resistance against commercial standards. Photostability was evaluated by repeated confocal scanning of GA-fixed cells. After 100 scans, PK Mito 590 FIX and MTR retained approximately 73% of their initial fluorescence, whereas PK Mito 647 FIX vastly outperformed MTDR by retaining approximately 94% versus 40%, respectively (Figures S16,S17). These data highlight the superior photobleaching resistance of the FIX probes, validating their exceptional suitability for continuous and high-resolution 3D imaging.

**Figure 3:**
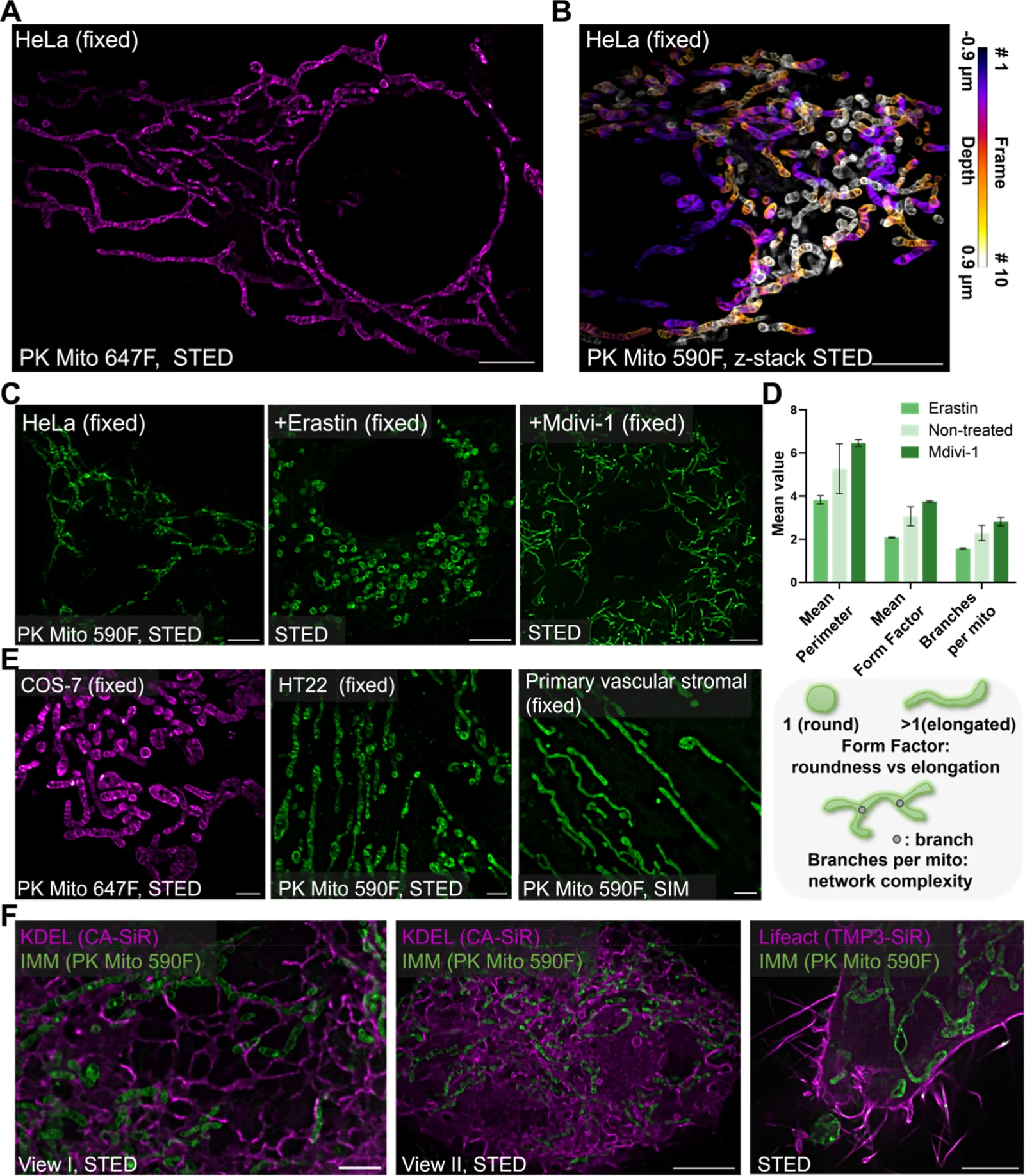
PK Mito 590/647 FIX enables STED/SIM imaging of mitochondria in fixed cells. (A) STED imaging of mitochondrial cristae of a fixed HeLa cell labeled with PK Mito 647F (300 nM, 15 min) and fixed with 2.5% GA. (Scale bar, 5 μm). (B) Z-stack STED imaging of mitochondrial networks in HeLa cells labeled with PK Mito 590F (300 nM, 15 min). The color code indicates z-position within a 2 μm range (Frame 1# to Frame 10#: −1.0 to +1.0 μm). (Scale bar, 5 μm). (C) Representative post-fixation STED imaging of HeLa cells after different drug stimulation. Left: STED image of untreated HeLa cells; Middle: STED image of HeLa cells treated with 20 μM erastin for 2 h; Right: STED image of HeLa cells treated with 40 μM Mdivi-1 for 1.5 h. All cells were stained with PK Mito 590F and fixed with 2.5% GA after drug treatment. (Scale bars, 5 μm). (D) Mitochondrial morphology analysis in nontreated cells, erastin-treated cells, and Mdivi-1-treated cells. (E) PK Mito FIX dyes labeled mitochondria in different types of cells. Cells were fixed with 2.5% GA. Left: post-fixation STED images of COS-7 cells labeled with PK Mito 647F. Middle: post-fixation STED images of HT22 cells labeled with PK Mito 590F. Right: post-fixation Hessian-SIM images of primary vascular stromal cells labeled with PK Mito 590F. (Scale bars, 2 μm). (F) Two-color post-fixation STED image of HeLa cells transfected with HaloTag-KDEL (left and middle) and TMP/eDHFR tag-Lifeact (right) and labeled with ligand-SiR and PK Mito 590F. (Scale bars: 2 μm, 5 μm, 2 μm).

To ensure that PK Mito FIX dyes could reliably monitor drug-induced mitochondrial morphological changes without introducing staining artifacts, we first evaluated their biocompatibility. Under routine labeling conditions (200–300 nM, ∼15 min), the probes induced no evident cytotoxicity or morphological alterations such as mitochondrial swelling or rounding (Figures S18–S20). Having established these nonperturbative conditions, we then utilized the probe to visualize distinct pharmacological responses in HeLa cells. Erastin, a known inducer of ferroptosis,^23^ triggered marked rounding and swelling of mitochondria in HeLa cells. In contrast, Mdivi-1, a potent dynamin-related protein 1 (Drp1) inhibitor of mitochondrial fission,^24^ rendered the mitochondria more elongated and network-forming (Figure 3C). Quantitative analysis of mitochondrial perimeter, form factor, and number of branches per mitochondrion^25^ further revealed that Erastin treatment increased mitochondrial perimeter and roundness while reducing the number of mitochondrial branches per mitochondrion, whereas Mdivi-1 exerted the opposite effects (Figure 3D). Morphological analysis of mitochondria following drug treatment was successfully performed on fixed cells, demonstrating that PK Mito FIX dyes hold promise as a new tool in the field of drug screening.

Furthermore, we assessed the staining efficiency of PK Mito 647/590 FIX across multiple cell lines, including COS-7 cells, mouse hippocampal neuronal HT22 cells, and primary vascular stromal cells (Figure 3E). Specifically, COS-7 cells were labeled with PK Mito 647 FIX, while HT22 cells and primary vascular stromal cells were stained with PK Mito 590 FIX. Owing to the high staining efficiency and cross-linking capacity of PK Mito FIX dyes, distinguishable cristae structures were clearly resolved in post-fixation STED imaging of COS-7 and HT22 cells. Beyond immortalized cell lines, we further explored the versatility of these dyes in primary cultures. In fixed primary vascular stromal cells, the probes enabled high-fidelity visualization of distinct mitochondrial structures using Hessian structured illumination microscopy (Hessian-SIM).^26,27^ Similarly, in neonatal cardiomyocytes and fibroblasts, PK Mito 590 FIX and PK Mito 647 FIX yielded robust mitochondrial labeling that proved fully compatible with both live-cell and post-fixation confocal and STED nanoscopy (Figure S21). Collectively, PK Mito FIX dyes demonstrate excellent compatibility with various cell lines, serving as a universal tool for mitochondrial labeling.

The combination of self-labeling tags and high-performance fluorophores has recently emerged as a common labeling strategy for super-resolution imaging.^28,29^ The staining and fixation protocol of PK Mito FIX dyes is also compatible with such protein-labeling strategies, allowing two-color recordings within cells. We expressed the F-actin marker Lifeact^30^ as a fusion protein with TMP-tag in HeLa cells and costained the cells with PK Mito 590 FIX and the deep-red fluorophore TMP3-Silicon rhodamine (TMP3-SiR).^31^ We were able to observe mitochondria being surrounded and enclosed by actin filaments at a peripheral region of the cell in the post-fixation STED imaging (Figure 3F). Similarly, HeLa cells expressing endoplasmic reticulum marker KDEL fused to HaloTag^32^ provided two-color recordings of the intricate structural organization and interaction between mitochondria and the endoplasmic reticulum (Figure 3F). Together, these results demonstrate that PK Mito FIX dyes serve as universal tools for mitochondrial labeling and are compatible with self-labeling protein systems for dual-color super-resolution imaging applications.

### Imaging of Fixed Mitochondria in Multicellular Systems

The PK Mito FIX series mitochondrial probes, characterized by their superior permeability and enhanced fixation performance, motivated us to further evaluate their imaging capabilities in multicellular samples. Patient-derived tumor-like cell clusters (PTCs),^33^ which are miniature in vitro versions of organs, possess significant potential for studying human diseases and elucidating their underlying pathogenic mechanisms. Four fixable mitochondrial dyes (PK Mito 647 FIX, PK Mito 590 FIX, PKMO FX, and MTR) were used to stain PTCs derived from sarcoma patients under identical conditions: 500 nM, 1.5 h incubation, followed by fixation with 2.5% GA. Confocal images of the samples pretreated with four dyes before fixation are presented in Figure S22. For the spheres of comparable size, PK Mito 647 FIX achieved a deeper penetration depth than PK Mito 590 FIX within the same staining period, confirming its better permeability in PTCs samples. Both dyes enabled clear visualization of filamentous mitochondrial architectures post-fixation. In contrast, the first-generation PKMO FX and the commercial MTR, despite adequate permeability, exhibited diffused mitochondrial signals after fixation, accompanied by prominent bright puncta, which are attributed to the aggregation of dye molecules that escaped the cross-linking within mitochondria. We further performed 3D reconstruction of z-stack images from a PK Mito 647 FIX-stained, GA-fixed PTCs spheroid (Figure 4A). Mitochondria displayed distinct spatial distributions across different layers, revealing the complex heterogeneity of the mitochondrial network throughout the whole organoid spheroid along the *Z*-axis (Figure 4B). We also evaluated the applicability of our probes in isolated mouse islet tissues. Interestingly, PK Mito 590 FIX worked better than 647 FIX in this tissue type, exhibiting satisfactory penetration efficiency. Representative confocal images of fixed mitochondria from different z-sections of a same isolated mouse islet are shown in Figure 4C. It should be noted that, in comparison with live islet imaging (Figure S23), the fixed islet inevitably showed a certain degree of shrinkage and alterations in mitochondrial morphology. Circled regions and line profiles indicate that the signal-to-background ratios can reach approximately 6.5×, demonstrating a high signal-to-noise ratio (Figure 4D). Together, the optimized permeability and fixation compatibility of PK Mito FIX dyes enable clear, artifact-minimized visualization of mitochondrial architecture within 3D samples. They successfully resolve mitochondrial heterogeneity across different layers of organoids and tissues, providing a reliable tool for advancing mitochondrial research in physiologically relevant models.

**Figure 4:**
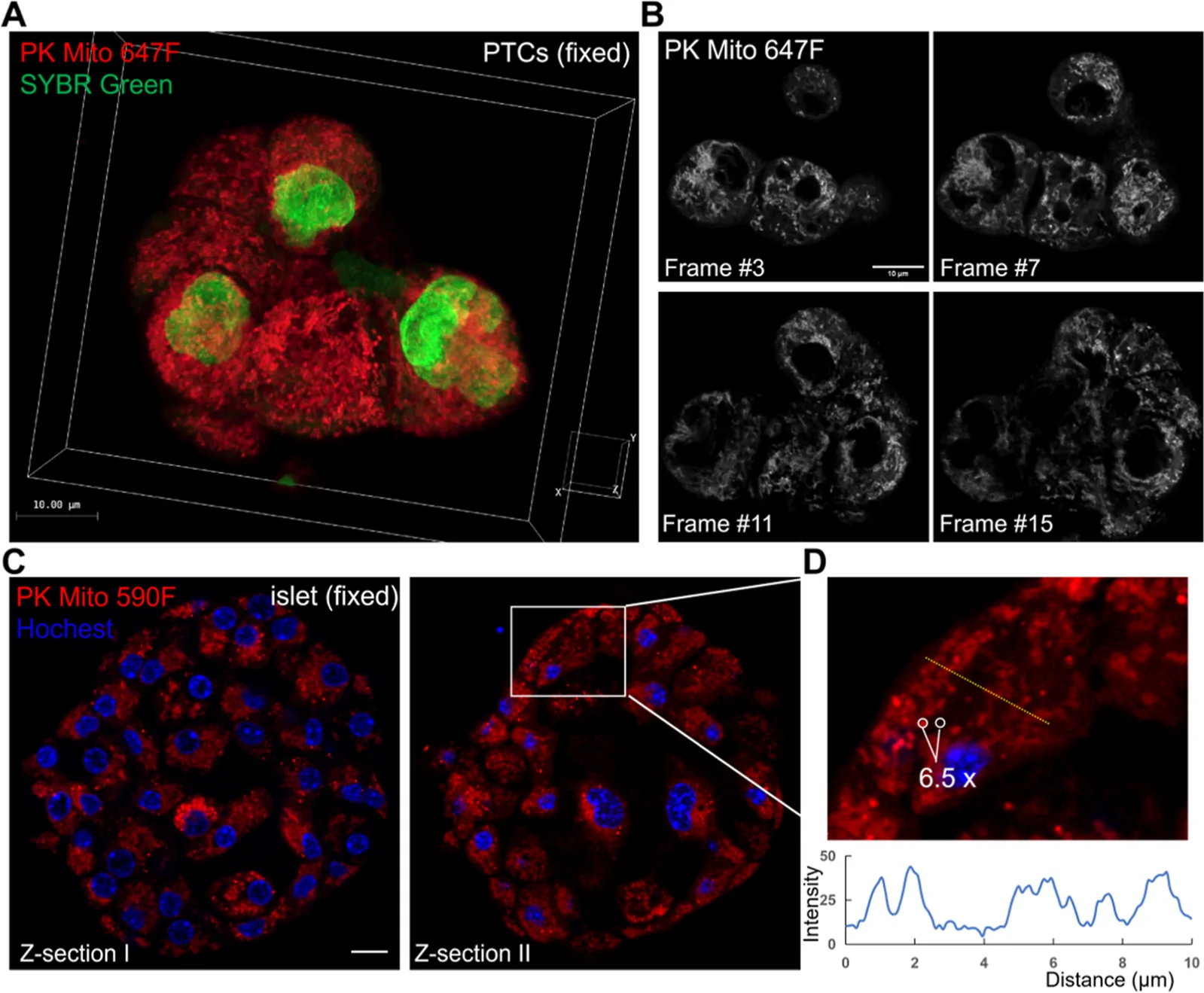
Imaging of fixed mitochondria in multicellular samples. (A) Z-stack confocal images and 3D reconstruction of patient-derived tumor-like cell clusters (PTCs) colabeled with PK Mito 647 FIX (red) and SYBR Green (green), followed by fixation with 2.5% GA. Scale bar, 10 μm. (B) Four different z-section images of mitochondria corresponding to frames #3, #7, #11, and #15 in (A). Scale bar, 10 μm. (C) Different z-section images of fixed mitochondria from the same isolated mouse islet tissue. Red: mitochondria, PK Mito 590 FIX. Blue: nucleus, Hochest. Scale bar, 10 μm. (D) Magnified view of the image in the white box area in C, and the plot profile along the yellow dashed line. Circled regions and line profiles highlight high signal-to-background ratios.

### Multicolor Super-Resolution Imaging of PK Mito 590/647 FIX Combined with Immunolabeling

Immunofluorescence staining stands as one of the most widely used approaches for the fluorescent labeling of target proteins. Its standard workflow generally involves chemical fixation of biological samples, followed by incubation with fluorophore-conjugated antibodies to specifically label the intended targets. Considering the good signal retention capability of PK Mito FIX dyes, we further explored whether these dyes are compatible with immunofluorescence staining protocols. HeLa cells were first stained with either PK Mito 590 FIX or PK Mito 647 FIX and then fixed with GA, permeabilized with Triton X-100, and subsequently immunostained with antibodies targeting diverse proteins or protein-binding toxins. For HeLa cells labeled with PK Mito 590 FIX, we performed immunolabeling of four distinct intracellular targets using Alexa 647-conjugated fluorescent secondary antibodies: specifically, mitochondrial outer membrane protein TOM20 (Figure 5A), mitochondrial DNA-binding protein TFAM (Figure 5B), microtubule protein tubulin (Figure 5C,H), and mitochondrial inner membrane protein COX IV (Figure 5D). Similarly, for HeLa cells labeled with PK Mito 647 FIX, three intracellular targets were immunolabeled using Alexa 555- or Alexa 488-conjugated fluorescent secondary antibodies: microtubule protein tubulin (Figure 5E), lysosome-associated membrane glycoprotein 1, LAMP1 (Figure 5F), and cytoskeletal protein F-actin (Figure 5G). By combining fixable mitochondrial probe labeling with immunolabeling, the interactions between mitochondria and various other intracellular structures can be conveniently imaged in a multiplexed manner.

**Figure 5:**
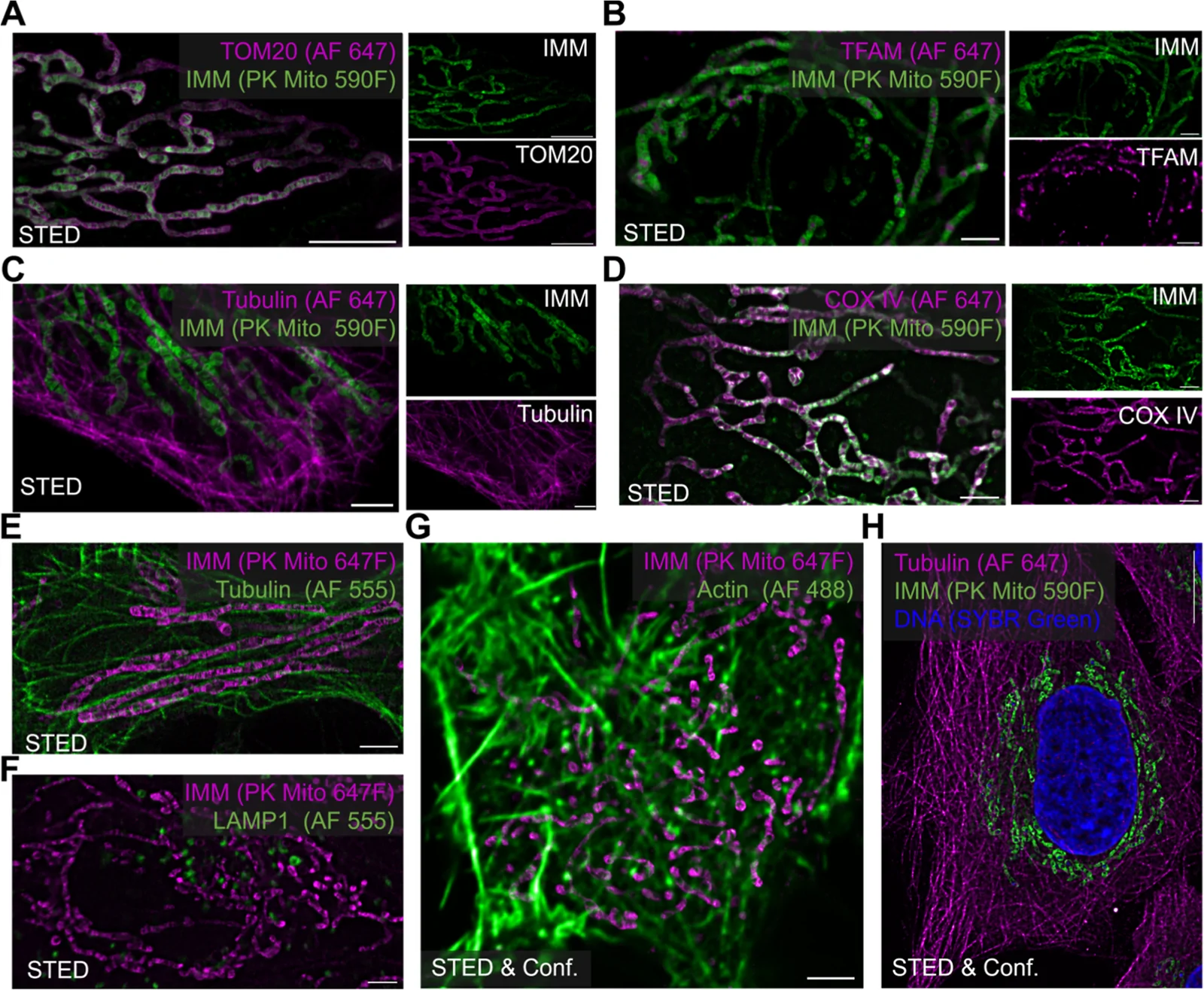
Multiplexed imaging using PK Mito 590*F*/647F combined with immunolabeling. (A–D) Dual-color STED images of GA-fixed HeLa cells stained with PK Mito 590F and coimmunostained with anti-TOM20 (A), anti-TFAM (B), anti-tubulin (C), and anti-COX IV (D). Left panels show composite overlays; right panels show individual split channels. Scale bars: 5 μm (A) and 2 μm (B–D). (E–G) Dual-color STED images of GA-fixed HeLa cells stained with PK Mito 647F and coimmunostained with anti-tubulin (E), anti-LAMP1 (F), and anti-actin (G). Scale bars, 5 μm. (H) STED imaging of a GA-fixed HeLa cell labeled with PK Mito 590F and SYBR Green, and immunolabeled for tubulin. Scale bar, 10 μm.

### PK Mito 590/647 FIX are Compatible with Expansion Microscopy

In addition to super-resolution microscopy, the superior retention efficiency after fixation encouraged us to apply PK Mito FIX dyes to expansion microscopy (ExM). ExM achieves nanoscale imaging by enlarging chemically fixed specimens within a swellable hydrogel, thereby rendering subdiffraction features resolvable on standard confocal microscopes.^34,35^ In most ExM workflows, fluorescent labeling is provided by immunofluorescence after chemical fixation.^36^ While such antibody-based approaches have been widely successful, they can be constrained by the accessibility of epitopes,^37^ the low spatial resolution due to the large size of antibodies,^38^ and the finite penetration of antibodies in densely packed or thick samples.^39^ Small-molecule probes therefore represent an attractive alternative;^40^ however, existing mitochondrial dyes such as MitoTracker fail to maintain adequate signal for cristae-level imaging after ExM processing.^41^ Given the excellent fixation properties and chemical stability of PK Mito 590/647 FIX, we hypothesized that these dyes would retain their fluorescence and mitochondrial localization throughout the harsh expansion protocol.

We thus adapted an iterative ultrastructure ExM (iU-ExM) protocol for this analysis.^42^ Live cells were stained with PK Mito dyes (500 nM) for 30 min, fixed with GA, embedded in a polyacrylate hydrogel, and, after polymerization and denaturation, expanded in water (Figure 6A). Notably, we employed a single round of expansion to visualize cristae and avoid further signal loss using the iU-ExM approach. Gel diameter measurements confirmed uniform 7.2× linear expansion (Figure 6B), with individual nuclear area measurements showing a 6.6-fold increase at the cellular level (Figures 6C and S24). Consistent with previous reports showing superior retention of ATTO647N compared to cyanine dyes,^43^ PK Mito 647 FIX (ATTO647N-based) retained 56% of its pregelation intensity, while PK Mito 590 FIX (Cy3.5-based) retained 25% (Figure 6D). Despite this differential signal retention, both PK Mito 590 FIX (Figure 6E) and PK Mito 647 FIX (Figure 6F) labeling enabled clear visualization of mitochondrial cristae patterns using confocal imaging. Remarkably, in U-2 OS cells with densely packed cristae that are barely resolvable using STED microscopy,^17^ expanded samples show resolved fine cristae structures in both confocal mode and STED mode (Figure 6G,H). Line profile analysis comparing STED and confocal imaging of the same expanded U-2OS cells showed comparable resolution between the two techniques (Figure 6H), showcasing a sufficiently expanded sample that is no longer dependent on super-resolution techniques to image the fine structure of IMM.

**Figure 6:**
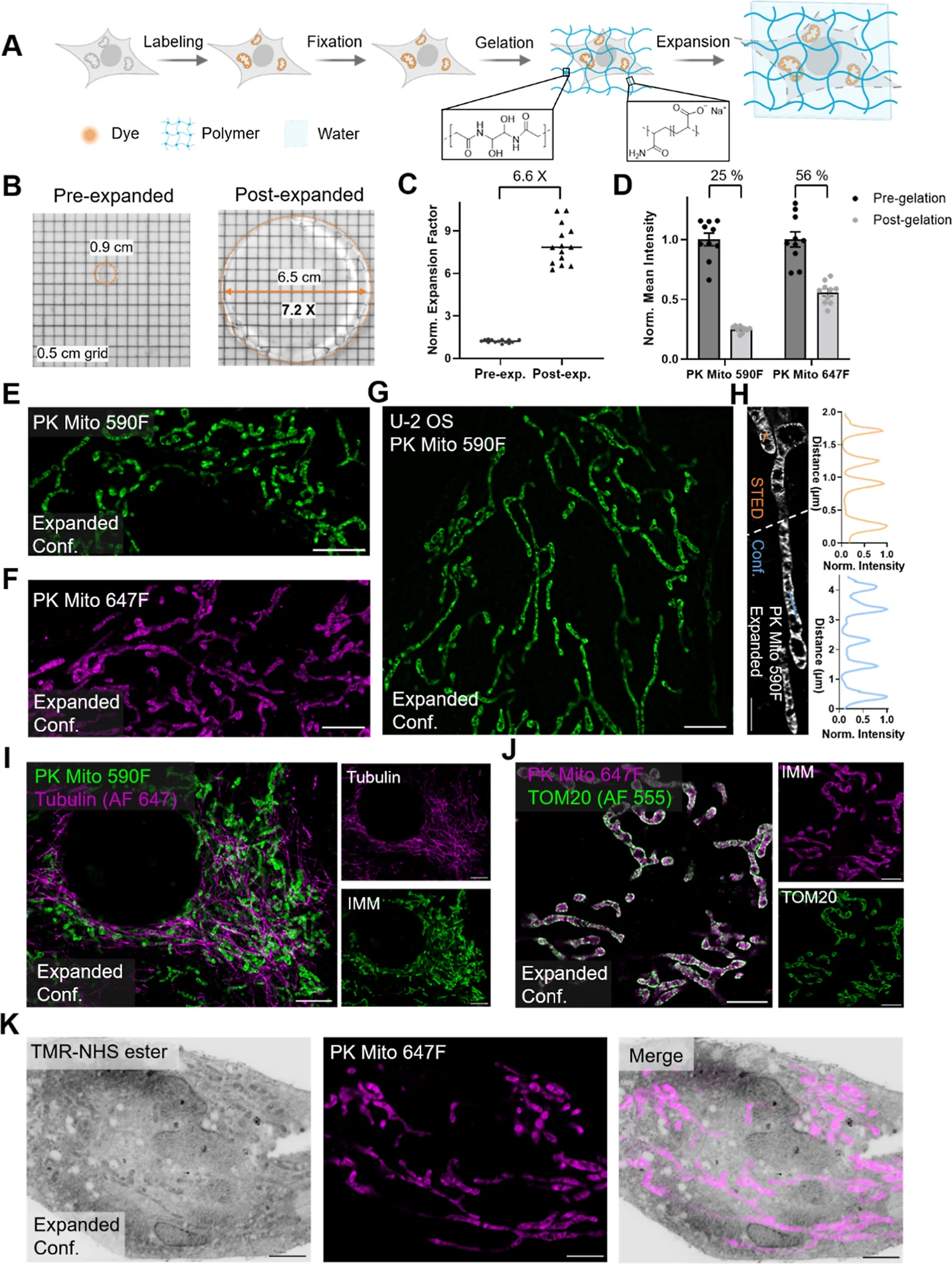
PK Mito 590F/647F are compatible with expansion microscopy. (A) Schematic workflow of expansion microscopy. (B) Representative gel photos before and after expansion on a 0.5 cm grid. (C) Quantification of nuclear area changes analyzed in ImageJ indicates an average linear expansion factor of ∼6.6×. (D) Quantification of normalized mean fluorescence intensity pre-gelation and post-gelation shows that PK Mito 590F retains 25% of its signal, while PK Mito 647F retains 56%. Error bars represent mean ± SEM. (E,F) Confocal imaging of HeLa cells stained with PK Mito 590F (E) and PK Mito 647F (F) after expansion. (Scale bars, 10 μm.) (G) Confocal imaging of U-2 OS cells stained with PK Mito 590 FIX after expansion. (Scale bar, 10 μm.) (H) U-2 OS cells stained with PK Mito 590F after expansion under confocal and STED microscopy. Line profiles show the high resolution for mitochondrial structures. (Scale bar, 5 μm.) (I) Dual-color imaging of PK Mito 590F (green, prestained) and anti-tubulin immunofluorescence (magenta, poststained) in expanded cells. (Scale bars, 10 μm.) (J) Dual-color imaging of PK Mito 647F (magenta, prestained) and anti-TOM20 immunofluorescence (green, poststained) in expanded cells. (Scale bars, 10 μm.) (K) Confocal images of expanded HeLa cells prelabeled with PK Mito 647F and postlabeled for pan-protein. (Scale bars, 10 μm.).

Furthermore, we showcase PK Mito FIX labeling with postexpansion immunostaining using PK Mito 590F/anti-tubulin pair (Figure 6I) and PK Mito 647F/anti-TOM20 pair (Figure 6J), demonstrating that the new PK Mito FIX dyes are fully compatible with postexpansion immunofluorescence for multicolor imaging of mitochondrial cristae alongside other cellular structures. We next assessed the compatibility of new probes with pan-ExM by combining PK Mito 647 FIX with TMR–NHS ester for pan-protein labeling,^44,45^ enabling the simultaneous imaging of both mitochondrial cristae and cellular context in expanded samples (Figure 6K). These results establish PK Mito 590 FIX and PK Mito 647 FIX as robust mitochondrial probes that withstand ExM processing while enabling cristae visualization on standard confocal microscopes.

## Conclusion and Discussion

In this study, we developed new fixable mitochondrial probes—PK Mito 590 FIX and PK Mito 647 FIX—which were rationally optimized from previous PKMO FX to achieve significantly improved performance. Through strategic amide-to-ester substitution and amine linker optimization, the probes now exhibit dramatically enhanced staining kinetics and signal-to-background ratios in live-cell staining while showing superior signal retention after fixation. PK Mito 590 FIX and 647 FIX enable high-quality visualization of mitochondrial ultrastructure across diverse biological samples, particularly in multicellular samples that were challenging to penetrate by PKMO FX, and are compatible with multiple imaging modalities. Furthermore, we demonstrated their compatibility with self-labeling tags, immunofluorescence protocols, and expansion microscopy, facilitating multicolor and multimodal imaging of mitochondria and other cellular structures.

Despite the advantages above, several promising directions for future work remain: 1. Spectral expansion: Developing yellow-green and NIR variants will broaden the color palette for multiplexed imaging of diverse cellular components. 2. Formaldehyde (FA) compatibility: Our current probes favor GA over FA due to the latter’s slower cross-linking kinetics.^46,47^ Future designs could therefore focus on fine-tuning structural optimization to enhance the compatibility with FA-based protocols. 3. Single-molecule localization microscopy (SMLM) adaptation: While ideal for STED and SIM, the probes lack the specific photoswitching behaviors required for SMLM, representing a key avenue for photophysical optimization. 4. Mechanistic exploration: Although increased lipophilicity facilitates our covalent fixation, the potential contributions of local microenvironmental effects or altered aggregation behavior warrant further mechanistic study.^48,49^ 5. Strategy generalization: The amide-to-ester substitution principle can potentially be generalized to improve the cellular permeability and fixability of fluorescent probes targeting other organelles. These future directions will expand the utility of our approach and potentially advance our understanding of cellular biology in physiological and pathological contexts.

## Supplementary Material



## Data Availability

The data supporting the findings of this study are available within the paper and its Supporting Information. Additional information and files are available from the corresponding author upon reasonable request.

## Notes

Materials and experimental procedures; cell culture, transfection, and primary mouse islet isolation; dye labeling and cell fixation protocols; live/fixed-cell confocal, high-content, and STED nanoscopy imaging; signal retention, chemical stability, photostability, and cell viability assays; expansion microscopy procedures; chemical synthesis and structural characterization (^1^H NMR and ESI-MS); Supplementary Tables S1, S2; and Supplementary Figures S1–S24 (PDF)
