# Supplementary material for: Mitochondrial Dyes with Balanced Permeability-Fixability for Live, Fixed, and Expanded Multicellular Imaging
Source: J Am Chem Soc. 2026 Sep 7;148(37):40556–67. doi: 10.1021/jacs.6c16881 (PMC13614188; doi:10.1021/jacs.6c16881)
Supplement: Supplementary file 1 [file ja6c16881_si_001.pdf]

Mitochondrial Dyes with Balanced Permeability-Fixability for Live, Fixed, and Expanded Multicellular Imaging

Jingting Chen[1#], Yue Ma[2#], Peng Chen[3], Biaoyi Yu[4], Ying Yu[5], Zhenzhen Zhang[6], Xiaomin Zhang[4], Buqing Ye[5], Jianzhong Jeff Xi[5], Xiaohong Peng[4], Zhixing Chen[1,2,3*]

1. College of Future Technology, Institute of Molecular Medicine, National Biomedical Imaging Center, Beijing Key Laboratory of Cardiometabolic Molecular Medicine, Peking University, Beijing 100871, China
2. Peking-Tsinghua Center for Life Science, Academy for Advanced Interdisciplinary Studies, Peking University, Beijing 100871, China
3. Genvivo Biotech (PuHaiJingShan), Nanjing 211800, China
4. School of Basic Medical Sciences, Shenzhen University Medical School, Shenzhen 518060, China
5. State Key Laboratory of Natural and Biomimetic Drugs, Institute of Molecular Medicine, Department of Biomedical Engineering, Peking University, Beijing 100871, China
6. Key Laboratory of Brain, Cognition and Education Sciences, Ministry of Education, China; Institute for Brain Research and Rehabilitation, and Guangdong Key Laboratory of Mental Health and Cognitive Science, South China Normal University, Guangzhou 510631, China

# These authors contributed equally.
To whom correspondence may be addressed: Zhixing Chen (zhixingchen@pku.edu.cn)

Table of contents

Materials and methods.. 2
Supplemental Figures..11
Chemical synthesis and characterization..33

Materials and methods

Absorption and fluorescence spectra

Stock solutions of PK Mito FIX dyes were prepared in DMSO at 1 mM and diluted with ethanol to 1 μM before measurement. Spectra were recorded at room temperature on a Tecan microplate reader (Tecan Austria GmbH) using Costar clear flat-bottom 96-well plates (Costar 3799). For absorbance measurements, PK Mito 590 FIX was scanned from 400-700 nm and PK Mito 647 FIX from 500-700 nm. For fluorescence measurements, PK Mito 590 FIX was excited at 530 nm with emission collected from 560–800 nm, while PK Mito 647 FIX was excited at 580 nm with emission collected from 610–800 nm. Normalized spectra are shown for clarity.

Cell culture and transfection

HeLa cells, COS-7 cells, and HT22 cells were cultured in plastic culture flasks in Dulbecco's Modified Eagle Medium (DMEM; Gibco, Thermo Fisher Scientific) supplemented with 10% fetal bovine serum (FBS; Gibco, Thermo Fisher Scientific), 1% 100 U/mL penicillin, 100 μg/mL streptomycin (Gibco, Thermo Fisher Scientific) at 37 °C and 5% CO_2. U-2 OS cells were cultured in plastic culture flasks in McCoy's 5A medium under the same culture conditions as described above.

Cells were plated in glass-bottom confocal dishes (Standard Imaging, China) for 24 h before staining and imaging. For transfection, cells were transfected transiently with corresponding plasmids using Lipo8000 reagent (Beyotime #C0533, China) according to the recommended proportion. After 6-10 h, residual transfection reagent was removed and the cells were washed with DMEM. The cells were maintained in fresh medium for another 18-24 h before staining and imaging.

Isolation and culture of mouse islets

Isolation and culture of mouse pancreatic islets were performed as previously described[1]. Briefly, islets were isolated from 8-week-old male mice raised under

specific pathogen-free (SPF) conditions. Following cervical dislocation, the pancreas was perfused with 2 ml of 0.5 mg/ml Collagenase P (11213873001, Roche), carefully dissected out, and then transferred to a centrifuge tube for digestion at 37℃ for 17 min. After digestion, the tissue was gently dissociated and mixed with ice-cold Hank's Balanced Salt Solution (HBSS, Gibco) supplemented with 10% FBS. Islets were allowed to settle on ice, resuspended, and the washing step was repeated with fresh HBSS/10% FBS. Finally, the islets were resuspended in HBSS/10% FBS and individually handpicked under a stereomicroscope. The selected islets were cultured overnight in RPMI 1640 medium (Gibco) supplemented with 10% FBS and 1% antibiotics (penicillin and streptomycin) at 37℃ in a humidified incubator with 5% CO_2.

Dyes labeling and fixation

In general, for HeLa cells, we recommend staining the cells with 200-300 nM PK Mito 590/647 FIX for 15 min. The cells were washed with fresh PBS buffer (pH =7.4) twice after staining and fresh medium was added into the dish for live-cell imaging. If the signal is noisy with dissatisfactory mitochondrial contrast, we recommend 15-min wash time in DMEM. After live-cell imaging of PK Mito FIX dyes, the cells were washed with preheated (37 °C) PBS buffer once and fixed with preheated (37 °C) 2.5% glutaraldehyde (GA, G916054, Macklin) for 15 min.

For imaging in neonatal primary cell models, pre-isolated neonatal cardiomyocytes (CMs) and fibroblasts (FBs) were plated in glass-bottom dishes before staining. For cardiomyocyte cultures, 0.1 mM 5-bromo-2′-deoxyuridine (BrdU; MCE, HY-15910) was included during culture to suppress the proliferation of non-cardiomyocytes. Cells were incubated with 200 nM PK Mito 590 FIX or PK Mito 647 FIX for 1 h at 37°C and 5% CO_2, washed with PBS, and imaged by confocal microscopy or STED microscopy as indicated. For post-fixation imaging, stained cells were fixed with pre-warmed 2.5% GA for 15 min, washed with PBS, and imaged after fixation.

Patient-derived tumor-like cell clusters (PTCs) derived from sarcoma patients were a kind gift from Jianzhong Jeff Xi lab. The PTCs were incubated with 500 nM

mitochondrial dyes (PKMO FX, PK Mito 590/647 FIX or PKMO) for 1.5 h at 37 °C and 5% CO_2. After staining, the samples were rinsed once with PBS buffer, and then fixed with 2.5% GA.

Imaging

Confocal and STED imaging: Confocal and STED images were acquired using an Abberior STEDYCON microscope (Abberior Instruments GmbH, Göttingen, Germany) equipped with a 100×/1.45 oil-immersion objective (Nikon, Tokyo, Japan). PK Mito 590 FIX was excited with a 561 nm laser, while PK Mito 647 FIX was excited using a 640 nm laser. Both dyes were depleted with a 775 nm pulsed depletion laser, with a gating window of 1–7 ns and a dwell time of 10 μs per pixel. STED images were deconvolved using Huygens professional or Lightbox software.

High-content imaging: High-content imaging was performed using an Opera Phenix high-content imaging system (PerkinElmer, Waltham, MA, USA) with a 20× air objective in widefield mode. PK Mito 590 FIX was excited with a 530-560 nm laser, and emission was collected through a 570-650 nm filter. PK Mito 647 FIX was excited with a 615-645 nm laser, and emission was collected through a 655-760 nm filter. Images were acquired using Harmony software.

Time-course labeling assay

HeLa cells were incubated with each candidate dye (300 nM) for 1, 3, 5, 10, or 20 min at 37°C. After incubation, cells were washed once with PBS and immediately imaged by confocal microscopy. For each channel-specific dye series (590 or 647), identical acquisition settings were maintained across all time points to enable direct comparison of signal intensities. During image processing in ImageJ, the contrast settings were kept constant across all time points for each individual dye to ensure fair visual comparison. Mitochondrial regions were segmented by threshold plugin in ImageJ, and mean fluorescence intensities were calculated by averaging measurements from at least three independent fields of view for each dye at each time point.

Mitochondrial colocalization assay

HeLa cells were first stained with PK Mito 590/647 FIX (300 nM, 10 min) and then stined with mitotracker dyes (200 nM, 10 min). PK Mito 590 FIX was paired with MitoTracker Deep Red FM (MTDR), while PK Mito 647 FIX was paired with MitoTracker Red CMXRos (MTR). After PBS washing, cells were imaged by confocal microscopy. Colocalization analysis was performed using ImageJ with the Coloc 2 plugin. Pearson's correlation coefficients (PCC) were calculated by ImageJ using the formula:

$$r = \frac{\sum_i (X_i - X_{avg})(Y_i - Y_{avg})}{\sqrt{\sum_i (X_i - X_{avg})^2 \sum_i (Y_i - Y_{avg})^2}}$$

where X_i and Y_i represent the intensity values of pixel in channels X and Y, respectively, and X_{avg} and Y_{avg} are the mean intensities of the corresponding channels. This analysis was performed on a pixel-by-pixel basis across the entire image field. Line profile analysis across representative mitochondrial structures was conducted to compare fluorescence intensity distributions between the two channels.

FCCP-induced mitochondrial fluorescence dissipation assay

HeLa cells were labeled with PK Mito 590 FIX or PKMO FX and washed with pre-warmed culture medium prior to live-cell imaging. Baseline mitochondrial fluorescence was established by acquiring initial frame using time-lapse confocal microscopy. To dissipate the mitochondrial membrane potential, 20 μM FCCP was added at the second frame, and time-lapse recording was continued under identical acquisition settings. Mean mitochondrial fluorescence intensity was quantified using ImageJ and normalized to the pre-treatment baseline intensity.

Retention and stability assay

Post-fixation signal retention: HeLa cells were labeled with 300 nM of each candidate dye for 10 min. Live cells were first imaged by confocal microscopy after identifying suitable fields of view. Without changing the field of view, cells were then fixed with 2.5% GA. After 10 min of fixation, the same fields were imaged again. For

each dye, this procedure was repeated three independent times. All image pairs (live/fixed) were acquired using identical acquisition parameters. For signal retention analysis, mitochondrial regions were segmented by threshold plugin in ImageJ, and the mean fluorescence intensities were measured. Signal retention ratio was calculated as the mean fluorescence intensity of fixed cells divided by that of live cells, with the final values representing the average of three independent experiments.

Detergent-resistant post-fixation retention: HeLa cells stained with PK Mito 590 FIX or PK Mito 647 FIX were fixed with 2.5% GA as described above. Confocal images were acquired before and after incubation with 5% Triton X-100 for 5 min. The mean mitochondrial fluorescence intensity was quantified using ImageJ, and signal retention was calculated relative to the pre-treatment intensity.

Chemical stability in PBS: Aqueous stability of PK Mito 590 FIX and PK Mito 647 FIX was assessed by HPLC-MS analysis (Agilent Technologies, Santa Clara, CA, USA). Each dye was prepared at an initial concentration of 150 μM in water and stored at room temperature. Aliquots were analyzed at 0 h, 26 h, 49 h, and 97 h. Chromatograms were monitored at the signature wavelength for each dye (590 nm or 650 nm), and corresponding mass spectra were recorded for each chromatographic peak.

Long-term post-fixation stability: HeLa cells were stained with 300 nM PK Mito 590 FIX or PK Mito 647 FIX for 10 min and then fixed with 2.5% GA. Samples were imaged on an Operetta high-content imaging system at 0.5 h, 2 h, 25 h, and 65 h after fixation. For each dye, three independent experiments were performed with seven fields imaged per time point. All images were acquired using identical acquisition settings across time points. Fluorescence intensity was quantified using ImageJ by measuring the mean intensity of entire images, and values were normalized to the 0.5 h time point to assess long-term signal stability.

Repeated-scan photostability assay

HeLa cells were stained with PK Mito 590 FIX, PK Mito 647 FIX, MitoTracker Red CMXRos, or MitoTracker Deep Red FM and fixed with 2.5% GA as described above. A 30 × 30 μm field was scanned continuously for 100 frames by confocal microscopy using unchanged acquisition settings within each dye-control pair. Mean mitochondrial fluorescence was quantified in ImageJ and normalized to frame 1.

CCK-8-based cell viability assay

Cells were seeded into 96-well plates (Zhenxuan, 11514) and allowed to adhere before dye treatment. PK Mito 590 FIX or PK Mito 647 FIX was diluted in complete culture medium to final concentrations of 0, 0.5, 1.0, 2.0, and 5.0 μM. Cells were incubated with the dyes for 0.5, 1, 3, or 6 h, followed by cell viability measurement using a Cell Counting Kit-8 assay (CCK-8; Beyotime, C0038) according to the manufacturer's instructions. Absorbance was measured at 450 nm using a microplate reader, and relative cell viability was calculated by normalizing each treatment group to the untreated control.

Mitochondrial morphology assessment

HeLa cells were incubated with PK Mito 590 FIX or PK Mito 647 FIX at concentrations of 0.25, 0.5, 1, or 2 μM for 0.5, 1, 2, or 3 h. Cells were subsequently imaged by STED microscopy, and images were systematically evaluated for signs of mitochondrial structural alterations, such as rounding or swelling.

Fluorescence imaging of islets

For live-cell imaging of pancreatic islets, intact islets cultured overnight post-isolation were hand-picked under a stereomicroscope and incubated for 1 hour at 37°C in Krebs-Ringer bicarbonate buffer (KRBB) solution containing 3 mM glucose and 200 nM PK Mito 590 FIX. The KRBB solution was composed of 125 mM NaCl, 5.9 mM KCl, 2.56 mM $CaCl_2$, 1.2 mM $MgCl_2$, 1 mM L-glutamine, 25 mM HEPES, and 0.1 % BSA. The stained islets were then seeded onto a 35-mm glass-bottom confocal dish and cultured for 24 hours in RPMI 1640 medium supplemented with 10% FBS and

antibiotics to facilitate adhesion. Subsequently, the medium was removed, and the islets were washed twice with pre-warmed KRBB containing 3 mM glucose. They were then stained with Hoechst 33342 (YK4114517, Thermo Fisher, diluted 1:1000) for nuclear labeling, incubated at 37°C for 30 minutes, and washed twice again with the same glucose-containing KRBB. Fluorescence images were acquired using a spinning-disc confocal microscope system built on an inverted Olympus IX83 microscope frame, equipped with a Yokogawa scanning unit and a 100 x oil immersion objective lens (Olympus). For post-fixation imaging, following the initial live-cell acquisition, the same islets were fixed with pre-warmed (37°C) glutaraldehyde for 30 minutes and then re-imaged using identical parameters.

Immunolabeling

HeLa cells were stained with 500 nM PK Mito 590/647 FIX for 15 min at 37°C and 5% CO_2. Before fixation, cells were washed with pre-warmed (37 °C) PBS once and then fixed by immersion with pre-warmed 2.5% GA for 15 min. The fixative was washed by PBS buffer three times after fixation. Cells were permeabilized with commercialized permeabilization buffer (Triton X-100, P0096, Beyotime) for 2 min and washed once with PBS. Cells were then incubated with blocking buffer (QuickBlock™, P0260, Beyotime) for 1 h at room temperature. Cells were then incubated with rabbit-derived primary antibodies including anti-TOM20; anti-tubulin; anti-COX IV; anti-TFAM; anti-LAMP1(1:75) overnight at 4 °C followed by washed with PBS buffer for 5 min x 3 times. Then the cells were incubated with secondary antibodies (goat anti-rabbit) with Alexa 647 fluorophore (Beyotime, P0180, 1:100) or AF555 fluorophore (Immunoway, RS3411) for 1h. Samples were washed with PBS 3 times, post-fixed with 2% GA and recorded under STED microscope the same day.

Expansion microscopy

Procedure	Reagent Name	Abbreviation	Manufacturer	Cat.

Fixation	2.5 % Glutaraldehyde	GA	Macklin	G916054
	8 % Paraformaldehyde	FA	Huayueyang biotech	HYJYKY6201
Polymerization	Acrylamide	AA	Energy Chemical	W610515
	Sodium acrylate	SA	BIDE	BD151354
	N,N'-(1,2-Dihydroxyethylene) bisacrylamide	DHEBA	Leyan	LY-TRCD493225
	N,N,N',N'-Tetramethylethylenediamine	TEMED	Energy Chemical	W820005
	Ammonium persulfate	APS	Energy Chemical	M0113816
Denaturation	20 % Sodium dodecyl sulfate solution	SDS	HARVEYBIO	SR5491
	Sodium chloride	NaCl	TGREAG	112008
	1M Tris solution, pH = 6.8	Tris	Double-helix biotech	P20211

Table S1. Expansion Microscopy Reagents
Key chemicals required for specimen fixation, hydrogel formation, and protein denaturation in the ExM protocol.

Procedure	Components	Solvent	Storage
**Anchoring solution**	1.4 % FA	1 x PBS	4 °C
	2 % (w/v) AA		
**Expansion monomer solution**	10 % (w/v) AA	1 x PBS	4 °C
	19 % (w/v) SA		
	0.1 % (w/v) DHEBA		
	0.25 % (v/v) TEMED		4 °C<br>Add when use
	0.25 % (w/v) APS		
**Denaturation buffer**	200 mM SDS	ddH_2O	RT
	200 mM NaCl		
	50 mM Tris		

Table S2. Expansion Microscopy Solution Preparation
Detailed reagent composition and storage conditions for ExM. TEMED and APS must be added immediately before use.

Sample Preparation and Gelation: Cells were cultured on 9 mm coverslips until

adherent, stained with 500 nM dye for 30 min, and rinsed with PBS. Samples were fixed in 2.5% glutaraldehyde (GA) for 15 min at room temperature and washed three times with PBS. Fixed samples were then incubated in the anchoring solution (Table S2) at 37°C for 3 h.

For gel embedding, coverslips were placed in a gelation chamber and overlaid with freshly prepared expansion monomer solution (Table S2). After 15 min on ice, polymerization was completed at 37°C for 45 min.

Denaturation and Expansion: Gel-embedded samples were immersed in denaturation buffer (Table S2) and gently shaken until the coverslips detached. Gels were transferred to fresh denaturation buffer and incubated at 85°C for 1.5 h. Following denaturation, gels were expanded by sequential exchanges of deionized water every 20–30 min until reaching a plateau (approximately 6–7× linear expansion).

Immunolabeling: Following expansion, gels were blocked with QuickBlock™ Blocking Buffer (P0260, Beyotime) for 15 min at room temperature. Gel pieces were then incubated overnight at 4°C with rabbit-derived primary antibodies including anti-TOM20 and anti-tubulin (1:200), followed by washing three times in PBS for 15 min. Secondary antibody labeling was performed using goat anti-rabbit antibodies conjugated with Alexa Fluor 647 (Beyotime, P0180, 1:200) or Alexa Fluor 555 (Immunoway, RS3411, 1:200) for 2 h. Gels were then washed three times in PBS for 15 min

NHS ester pan-staining: After immunolabeling, gels were incubated for 2 h with 25 μg/mL NHS ester-5-TAMRA (Solarbio, IT4010) dissolved in 100 mM sodium bicarbonate solution on a rocking platform at RT. Gels were then washed three times in PBS for 30 min per wash on a rocking platform. Before imaging, gels were transferred to deionized water and allowed to re-expand to the desired size.

Supplemental Figures

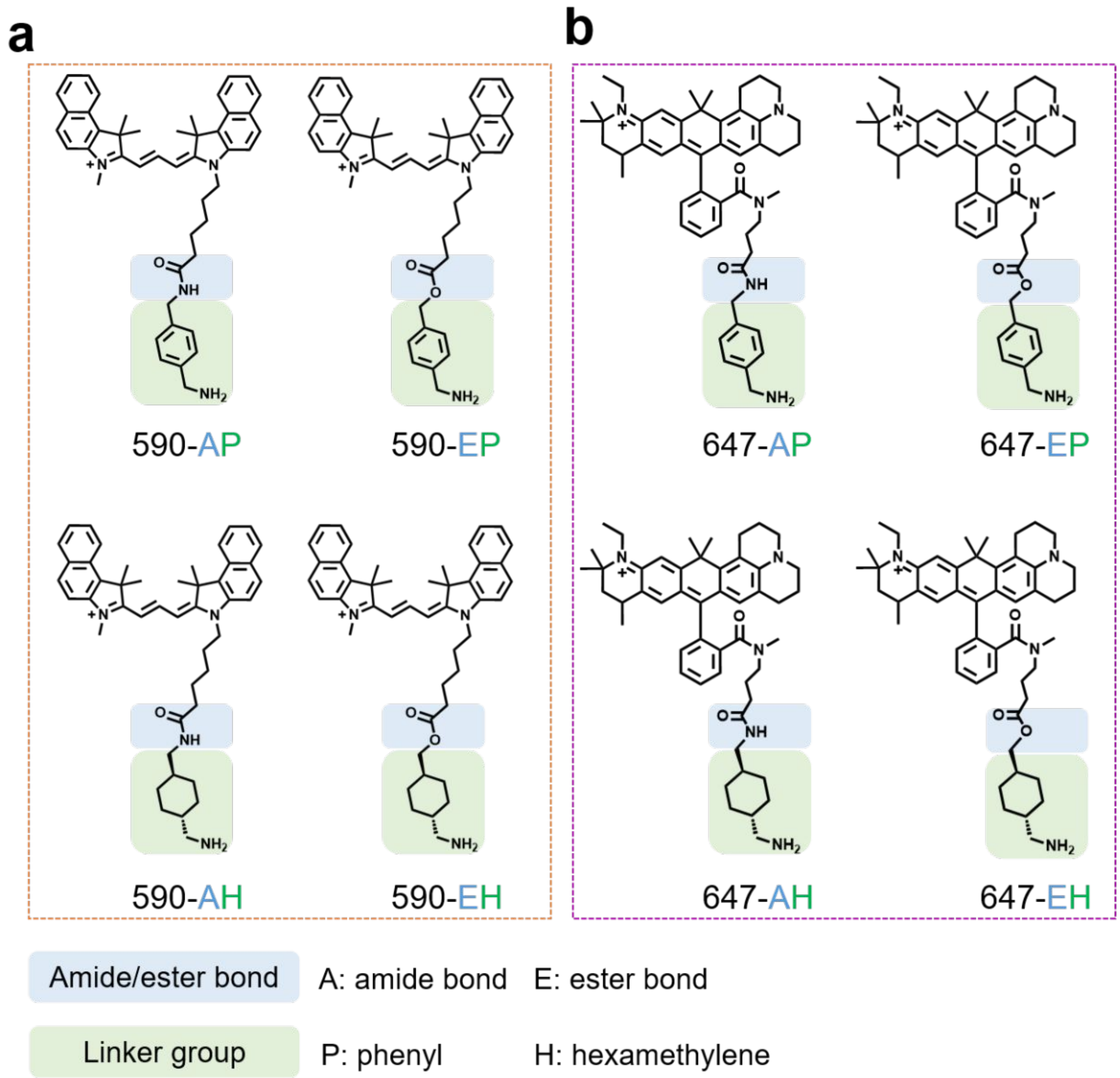

Fig. S1 Modular design matrix of fixable mitochondrial dye candidates.

a. Cy3.5-based candidates combining two linker types with two amine groups. Top row: amide linker (A) versus ester linker (E); left to right: phenylamine (P) and hexamethylene amine (H). The four variants are denoted as AP, EP, AH, and EH, respectively.

b. ATTO647N-based counterparts with the same linker–amine combinations, affording AP, EP, AH, and EH variants.

Color coding marks variable positions: pale blue, linker type (amide or ester); pale green, amine substituent (phenyl or hexamethylene). These candidates are evaluated for their impact on cellular labeling.

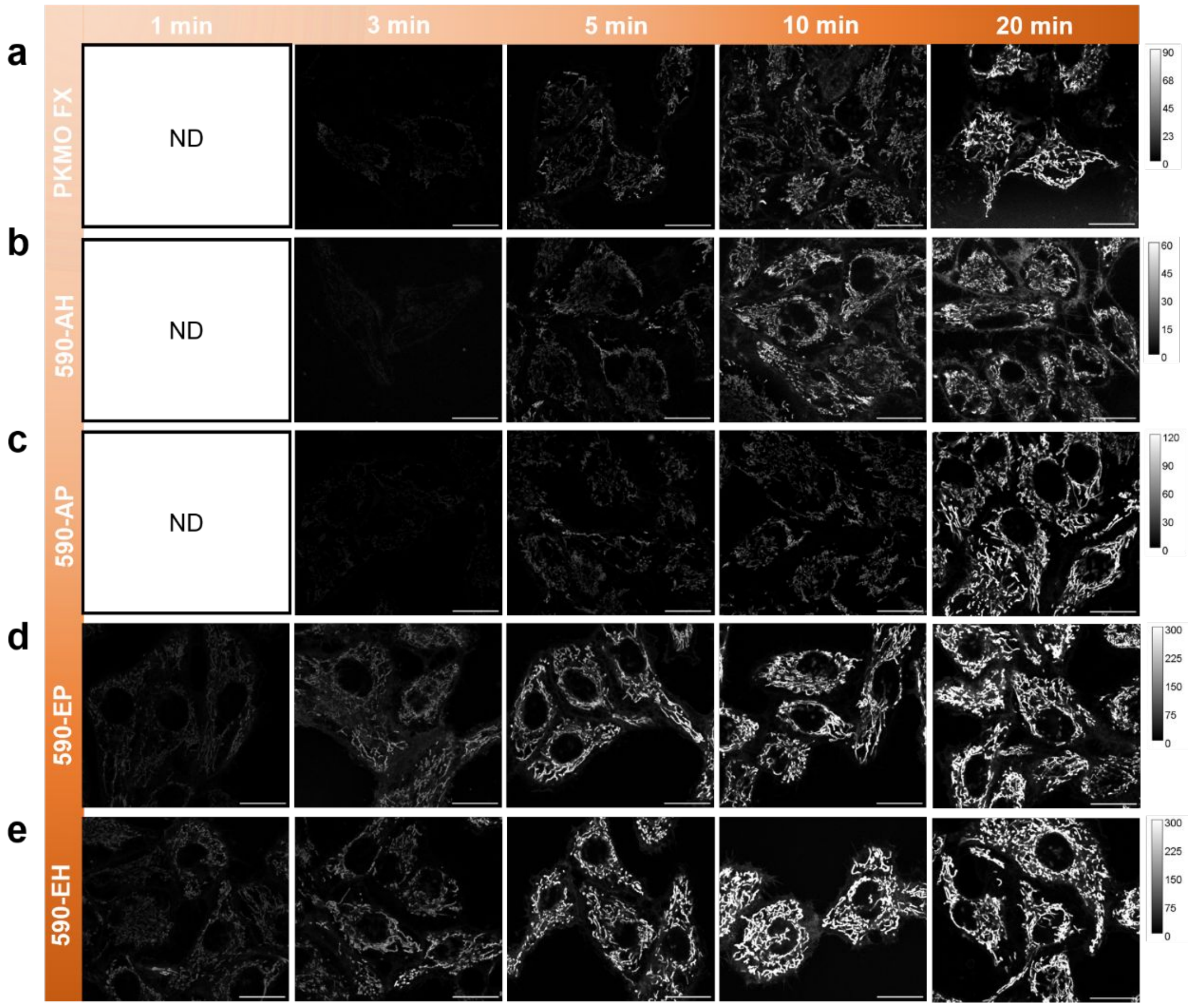

Fig. S2 Time-course labeling of Cy3.5-based fixable mitochondrial dye candidates in live cells.

a-e. Representative confocal images of HeLa cells labeled for 1, 3, 5, 10, and 20 min with five Cy3.5-channel candidates (rows, top to bottom): PKMO FX (1st generation), 590-AH, 590-AP, 590-EP, and 590-EH. Cells were incubated with each dye at 300 nM for the indicated time and then washed once with PBS immediately before imaging. Within each row, images were acquired and displayed with identical settings to allow visual comparison over time, and “ND” denotes “not detected” when the signal fell below the detection limit under these acquisition settings. The grayscale bars at right indicate the display range used for each row. Scale bars, 20 µm.

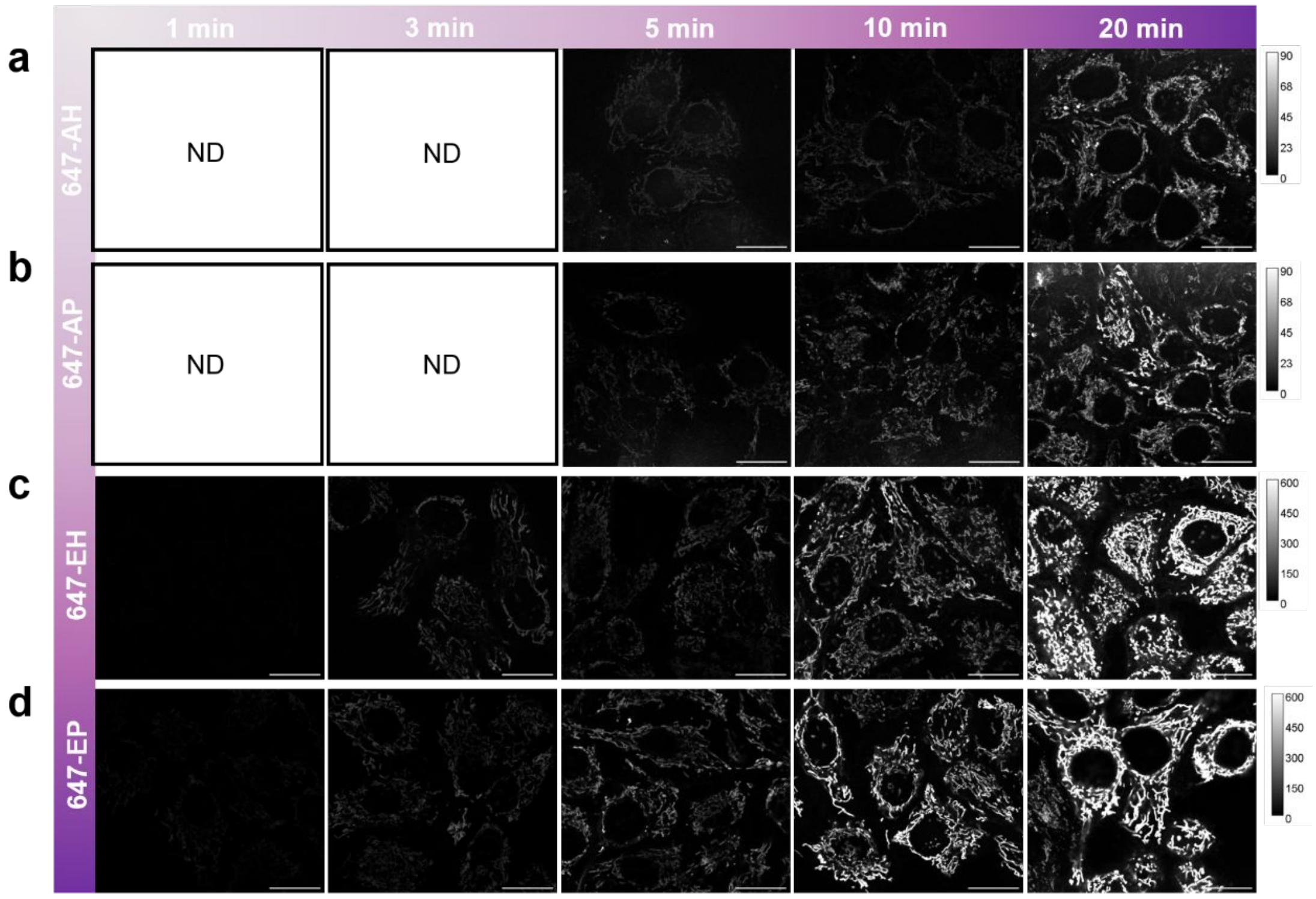

Fig. S3 Time-course labeling of ATTO647N-based fixable mitochondrial dye candidates in live cells.

a–e. Representative confocal images of HeLa cells labeled for 1, 3, 5, 10, and 20 min with four 647-channel candidates (rows, top to bottom): 647-AH, 647-AP, 647-EH, and 647-EP. Cells were incubated with each dye at 300 nM for the indicated time and then washed once with PBS immediately before imaging. Within each row, images were acquired and displayed with identical settings to enable visual comparison over time, and "ND" denotes "not detected" when the signal fell below the detection limit under these acquisition settings. The grayscale bars at right indicate the display range used for each row. Scale bars, 20 μm.

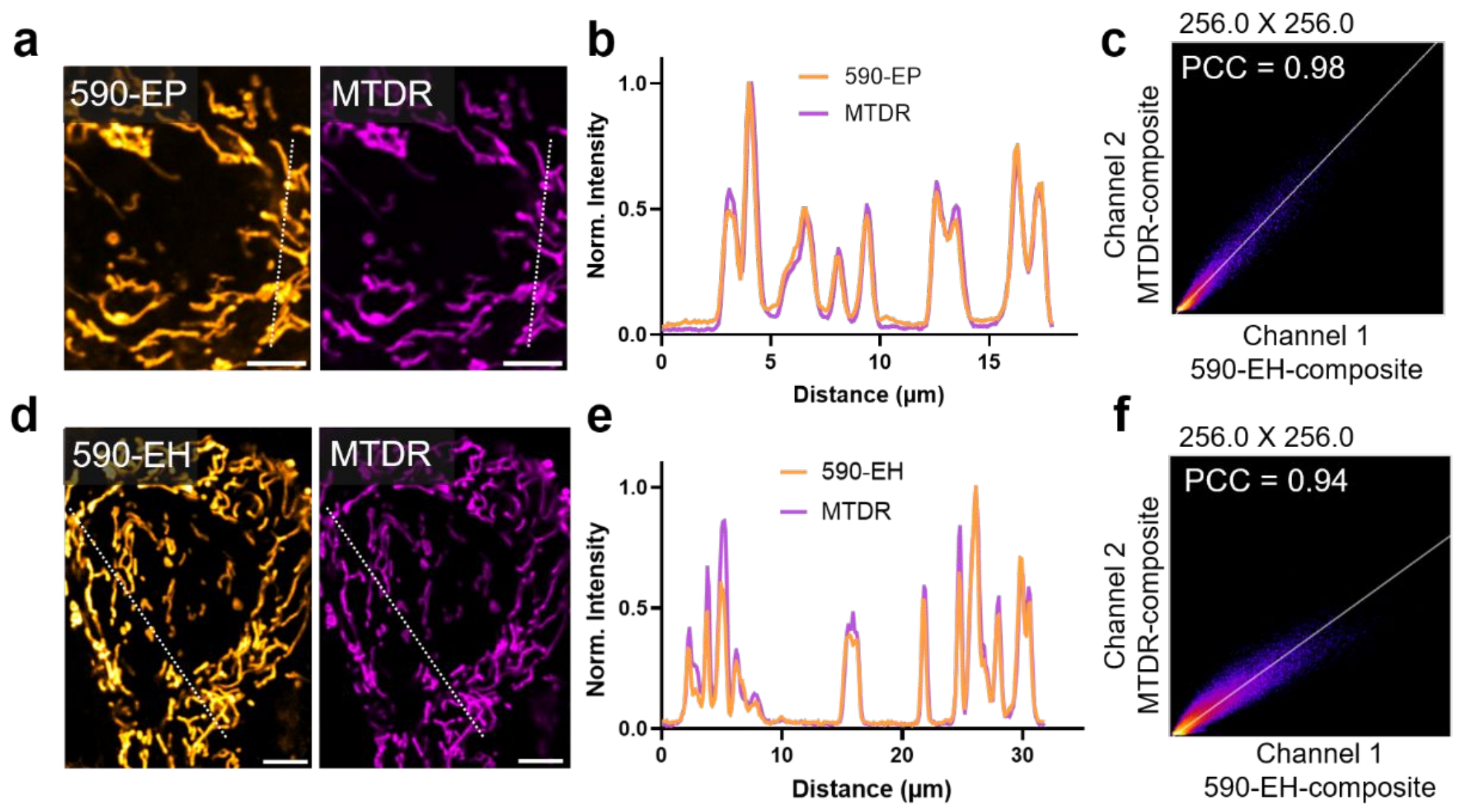

Fig. S4 Colocalization analysis of 590-EP and 590-EH with MitoTracker dyes.
a, d. Confocal images of mitochondria labeled with 590-EP (a) or 590-EH (d) and MitoTracker Deep Red (MTDR). Scale bars, 5 μm (a) and 10 μm (d).
b, e. Line intensity profile along the dotted lines in panel a (b) or d (e) , respectively.
c, f. Two-dimensional intensity correlation plot between 590-EP (a) or 590-EH (d) and MTDR signals with the Pearson's correlation coefficient (PCC).

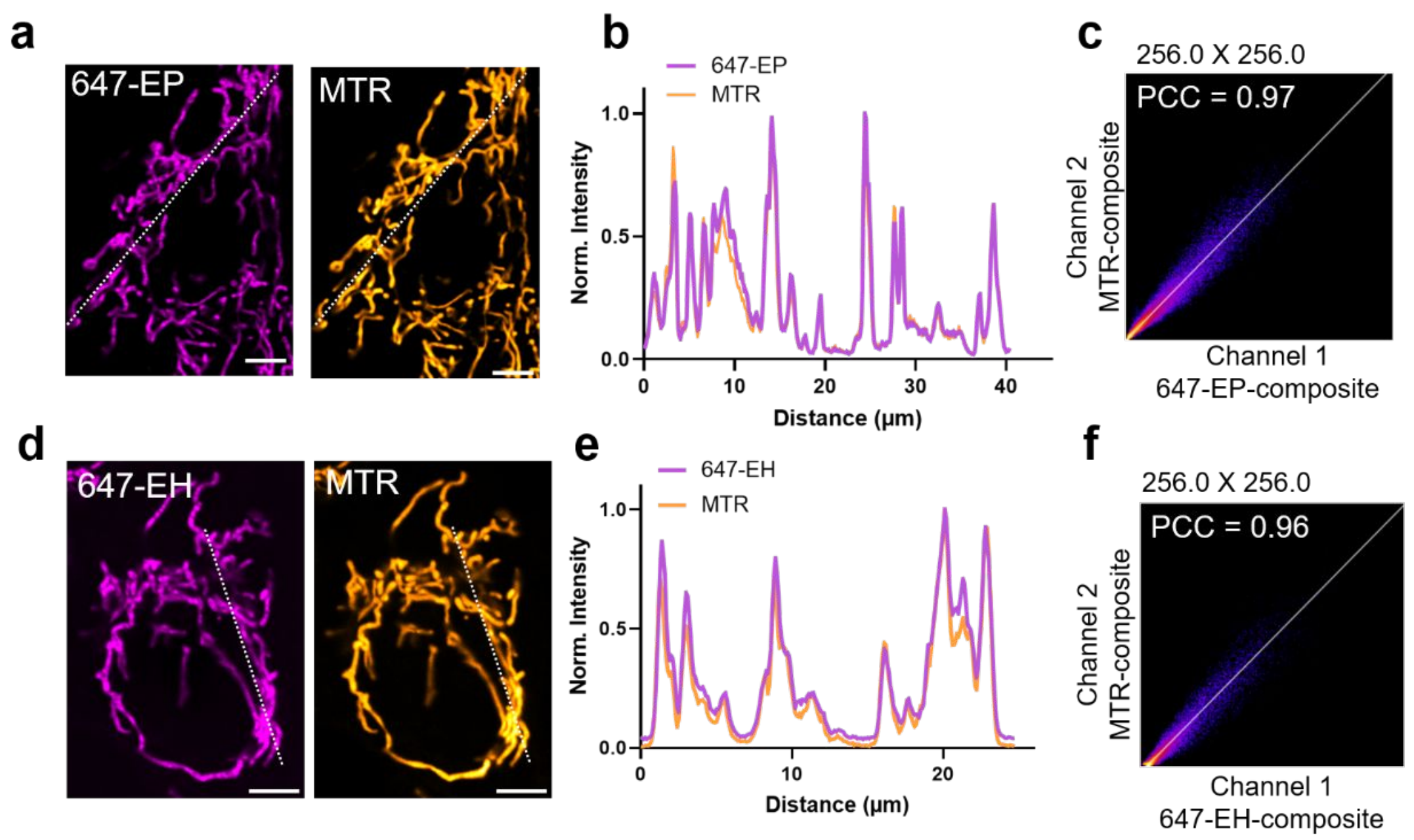

Fig. S5 Colocalization analysis of 647-EP and 647-EH with MitoTracker dyes.
a, d. Confocal images of mitochondria labeled with 647-EP (a) or 647-EH (d) and MitoTracker Red (MTR). Scale bars, 10 μm.
b, e. Line intensity profile along the dotted lines in panel a (b) or d (e) , respectively.
c, f. Two-dimensional intensity correlation plot between 647-EP (a) or 647-EH (d) and MTR signals with the Pearson's correlation coefficient (PCC).

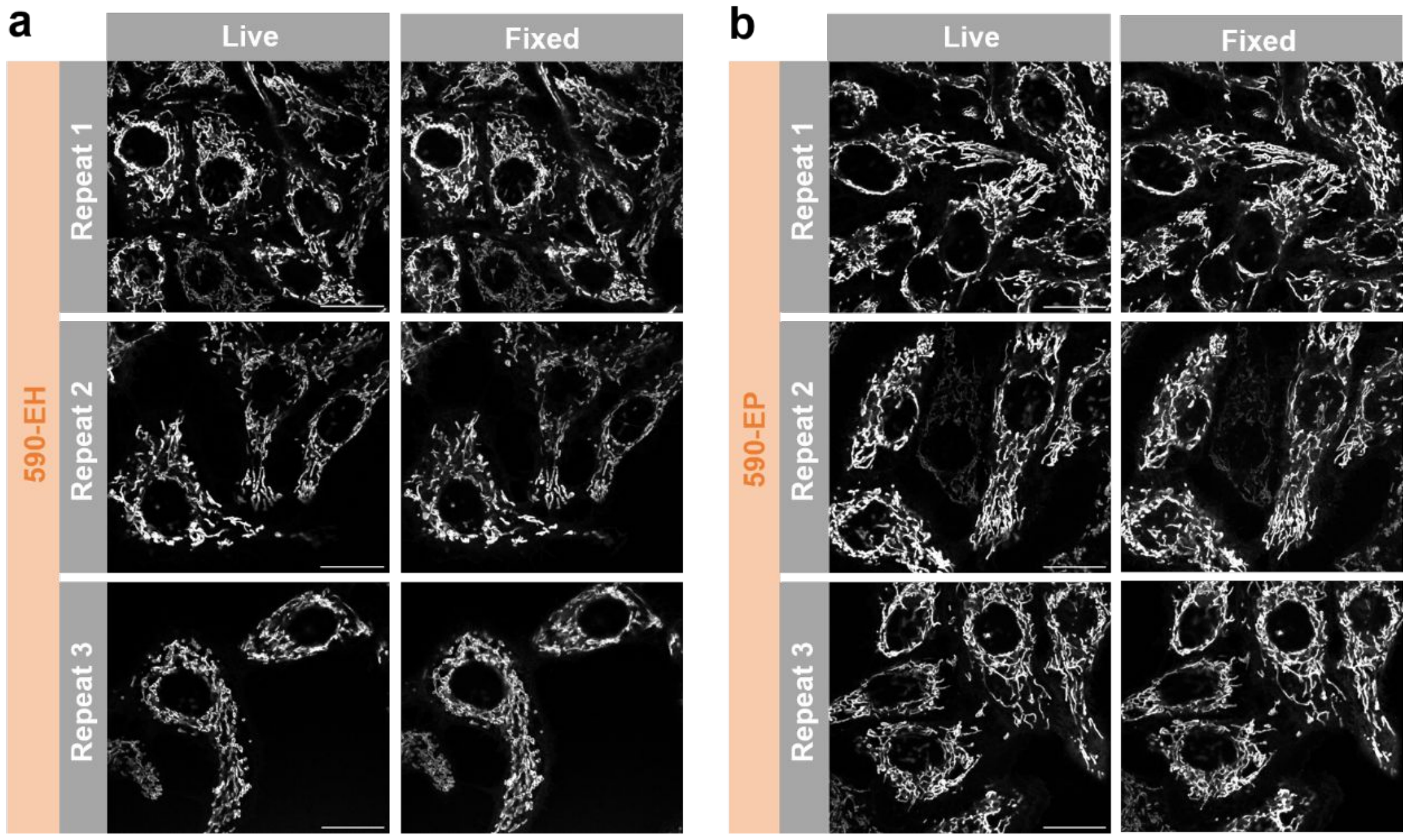

Fig. S6 Comparison of signal-to-noise and fluorescence retention of 590-EH and 590-EP after fixation

a. HeLa cells were labeled with 300 nM 590-EH for 10 min, then fixed with 2.5% glutaraldehyde (GA); images were acquired 10 min after fixation. Paired fields acquired in live and post-fixation conditions are shown for three independent experimental repeats. For each repeat, live/fixed image pairs were collected and displayed using identical acquisition and display parameters to enable direct assessment of signal-to-noise and fluorescence retention. Scale bars, 20 μm.

b. The same procedure was applied to 590-EP (300 nM, 10 min labeling; 2.5% GA fixation; imaging 10 min after fixation), and representative paired fields from three independent repeats are shown.

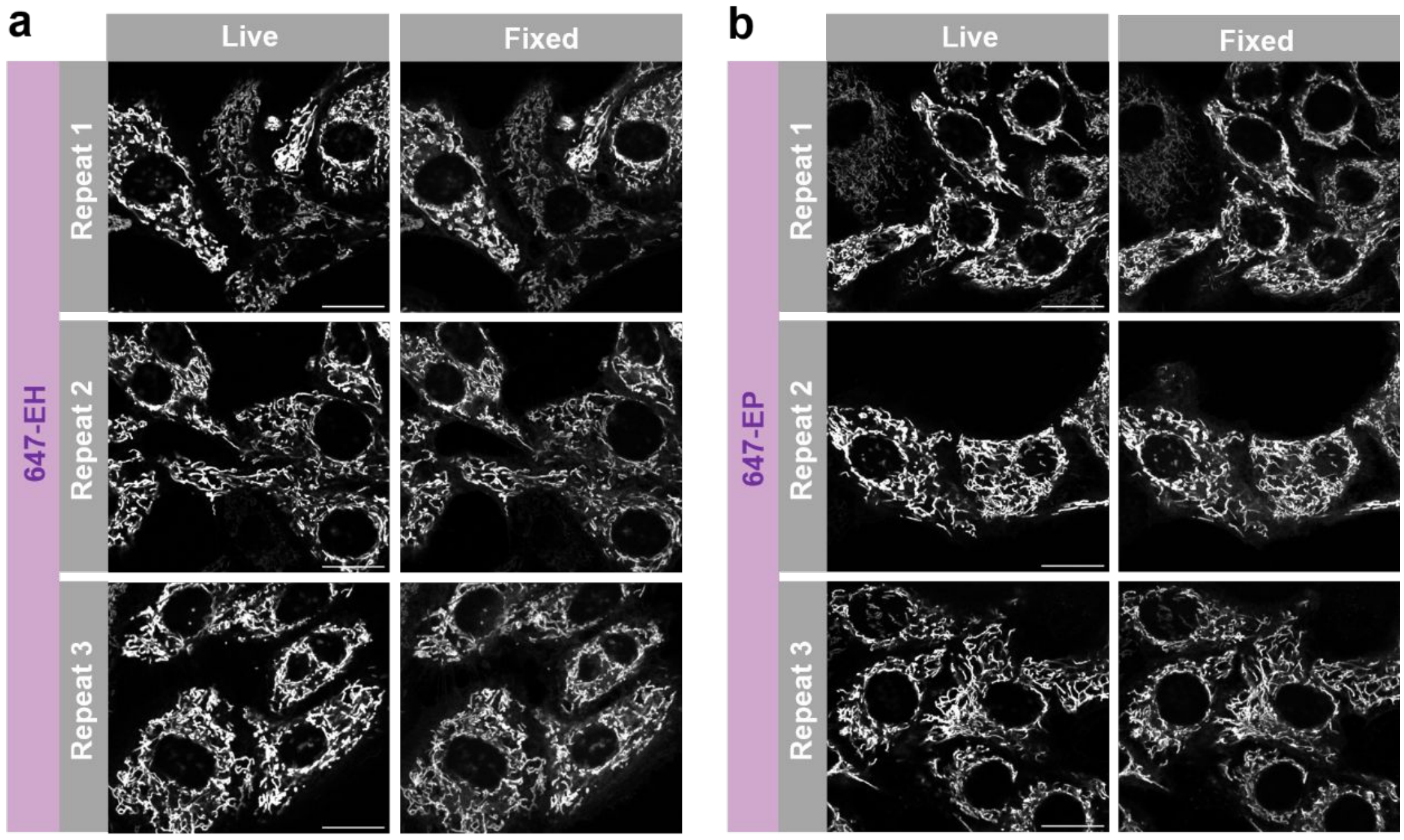

Fig. S7 Comparison of signal-to-noise and fluorescence retention of 647-EH and 647-EP after fixation

a. HeLa cells were labeled with 300 nM 647-EH for 10 min, then fixed with 2.5% glutaraldehyde (GA); images were acquired 10 min after fixation. Paired fields acquired in live and post-fixation conditions are shown for three independent experimental repeats. For each repeat, live/fixed image pairs were collected and displayed using identical acquisition and display parameters to enable direct assessment of signal-to-noise and fluorescence retention. Scale bars, 20 µm.

b. The same procedure was applied to 647-EP (300 nM, 10 min labeling; 2.5% GA fixation; imaging 10 min after fixation), and representative paired fields from three independent repeats are shown.

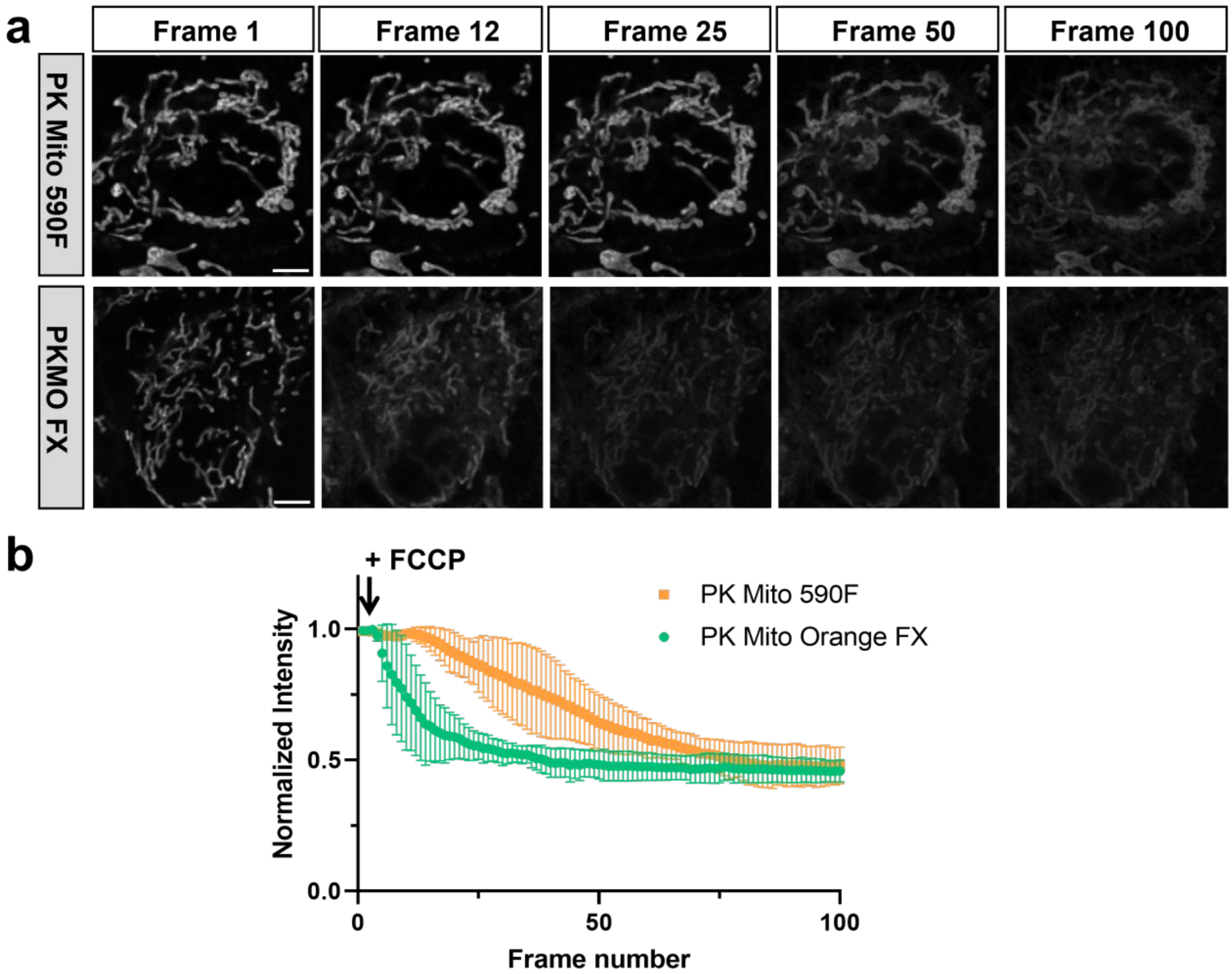

Fig. S8 FCCP-induced mitochondrial fluorescence dissipation of PK Mito 590 FIX and PKMO FX.

a. Time-lapse confocal images of HeLa cells stained with PK Mito 590 FIX or PKMO FX before and after treatment with 20 μM FCCP. Representative frames are shown. Scale bars, 5 μm.

b. Normalized mitochondrial fluorescence decay curves following FCCP addition. PK Mito 590 FIX showed slower signal dissipation than PKMO FX under the tested conditions.

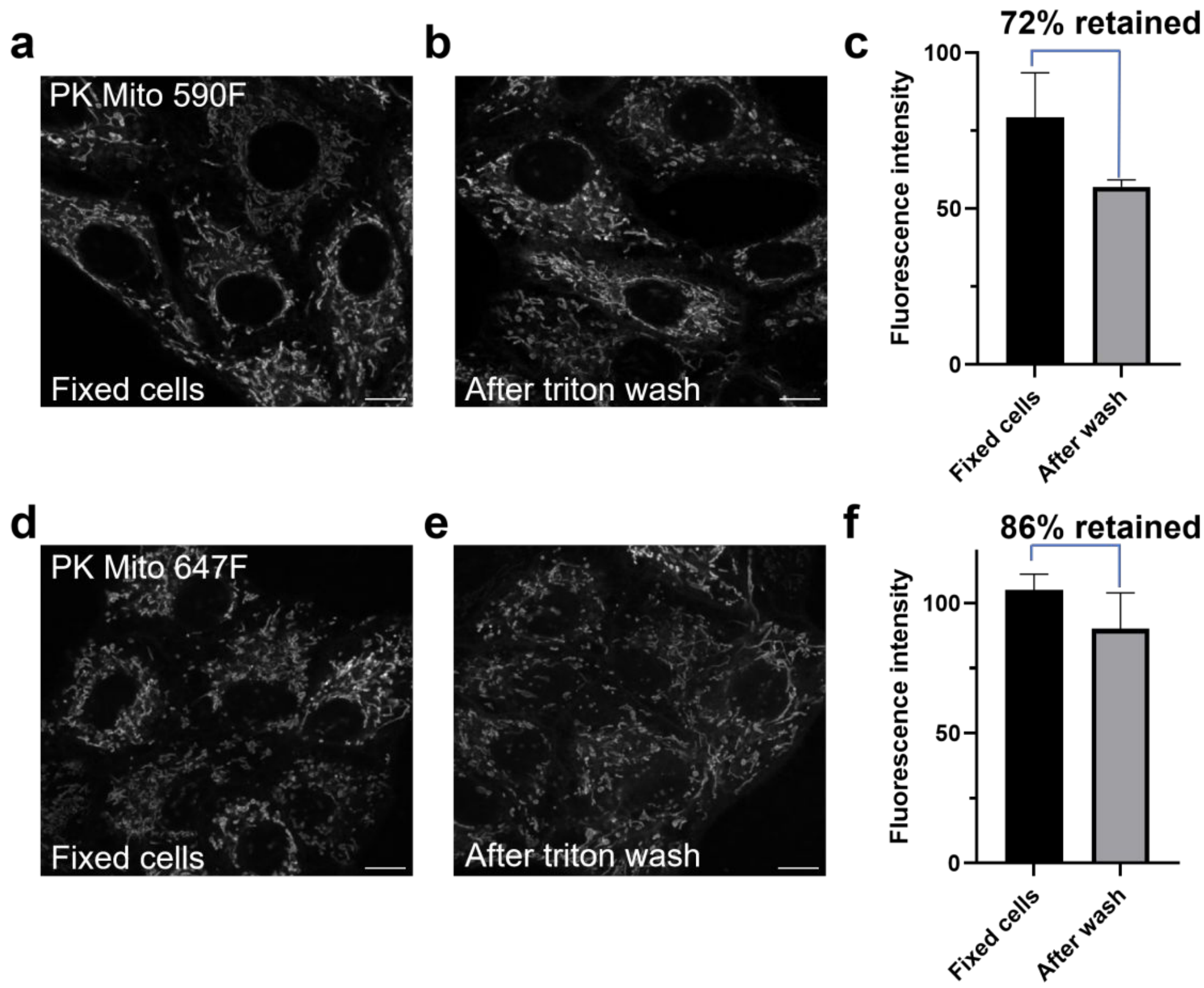

Fig. S9 Detergent-resistant retention of PK Mito FIX fluorescence after GA fixation.

a. Confocal image of GA-fixed HeLa cells stained with PK Mito 590 FIX.

b. Confocal image of the same sample after incubation with 5% Triton X-100 for 5 min.

c. Quantification of PK Mito 590 FIX fluorescence before and after Triton treatment; approximately 72% of the fluorescence was retained.

d. Confocal image of GA-fixed HeLa cells stained with PK Mito 647 FIX.

e. Confocal image of the same sample after incubation with 5% Triton X-100 for 5 min.

f. Quantification of PK Mito 647 FIX fluorescence before and after Triton treatment; approximately 86% of the fluorescence was retained. Data are presented as mean ± SEM.

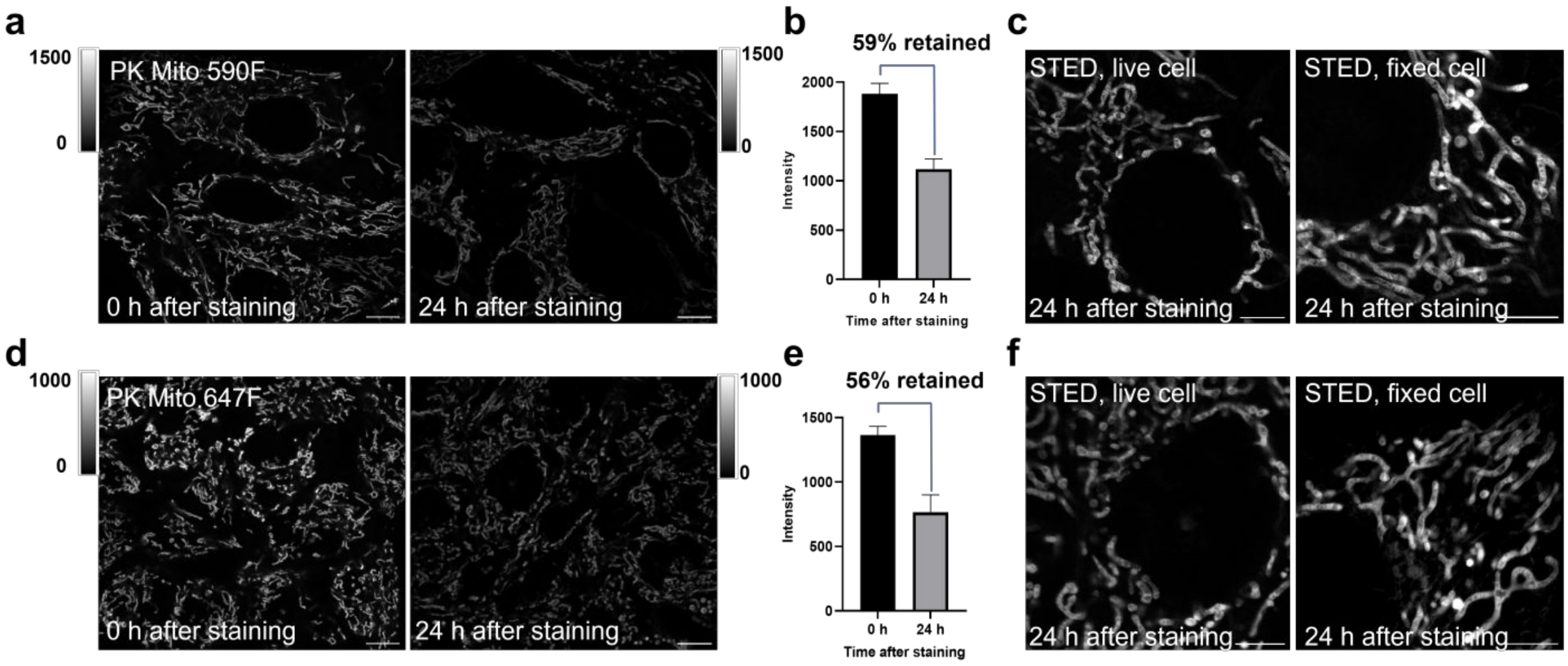

Fig. S10 Long-term cellular imaging and intracellular stability of ester-linked PK Mito FIX probes.

(a, d) Confocal images of live HeLa cells stained with PK Mito 590F (a) and PK Mito 647F (d), acquired 0 h (left) and 24 h (right)after dye washout. Scale bars: 10 µm.

(b, e) Quantitative analysis of the mean fluorescence intensity at 0 h and 24 h after staining. Specifically, 59% of the initial PK Mito 590F fluorescence signal (b) and 56% of the initial PK Mito 647F fluorescence signal (e) are retained over the 24-hour period.

(c, f) STED nanoscopy of live (left) and GA-fixed (right) HeLa cells stained with PK Mito 590F (c) and PK Mito 647F (f), acquired 24 h after dye washout. Scale bars: 5 µm.

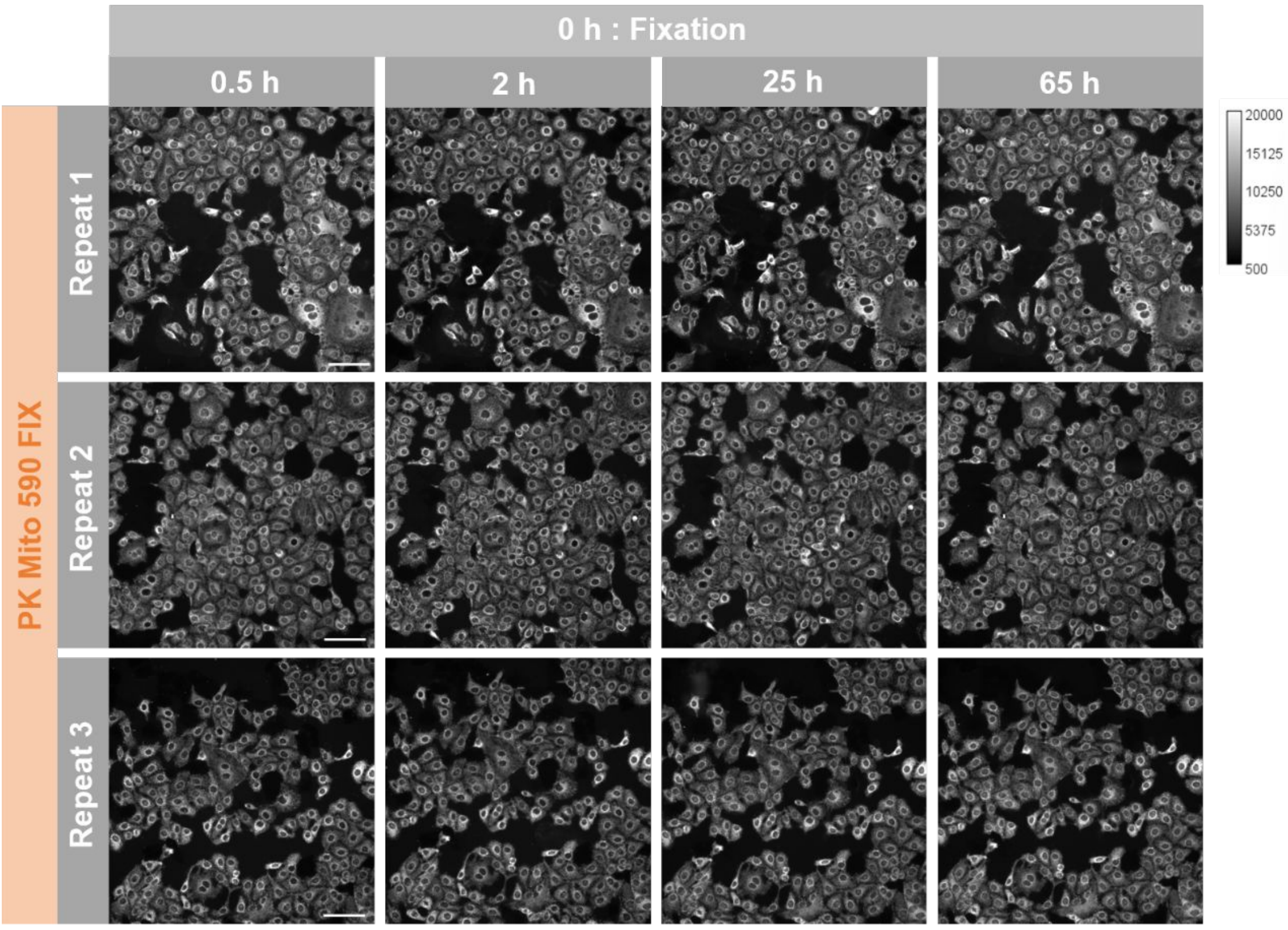

Fig. S11 Long-term fluorescence retention of PK Mito 590 FIX after fixation under high-content imaging.

HeLa cells were stained with 400 nM PK Mito 590 FIX for 10 min and then fixed with 2.5% glutaraldehyde (GA) at t=0 h. Representative fields from three independent experiments are shown, with one field per time point per experiment acquired on the Operetta high-content system at 0.5 h, 2 h, 25 h, and 65 h after fixation. For each experiment, seven fields were imaged, and the panels display a representative field captured and displayed with identical acquisition and display settings across time to enable direct comparison of fluorescence retention. Scale bars, 100 µm.

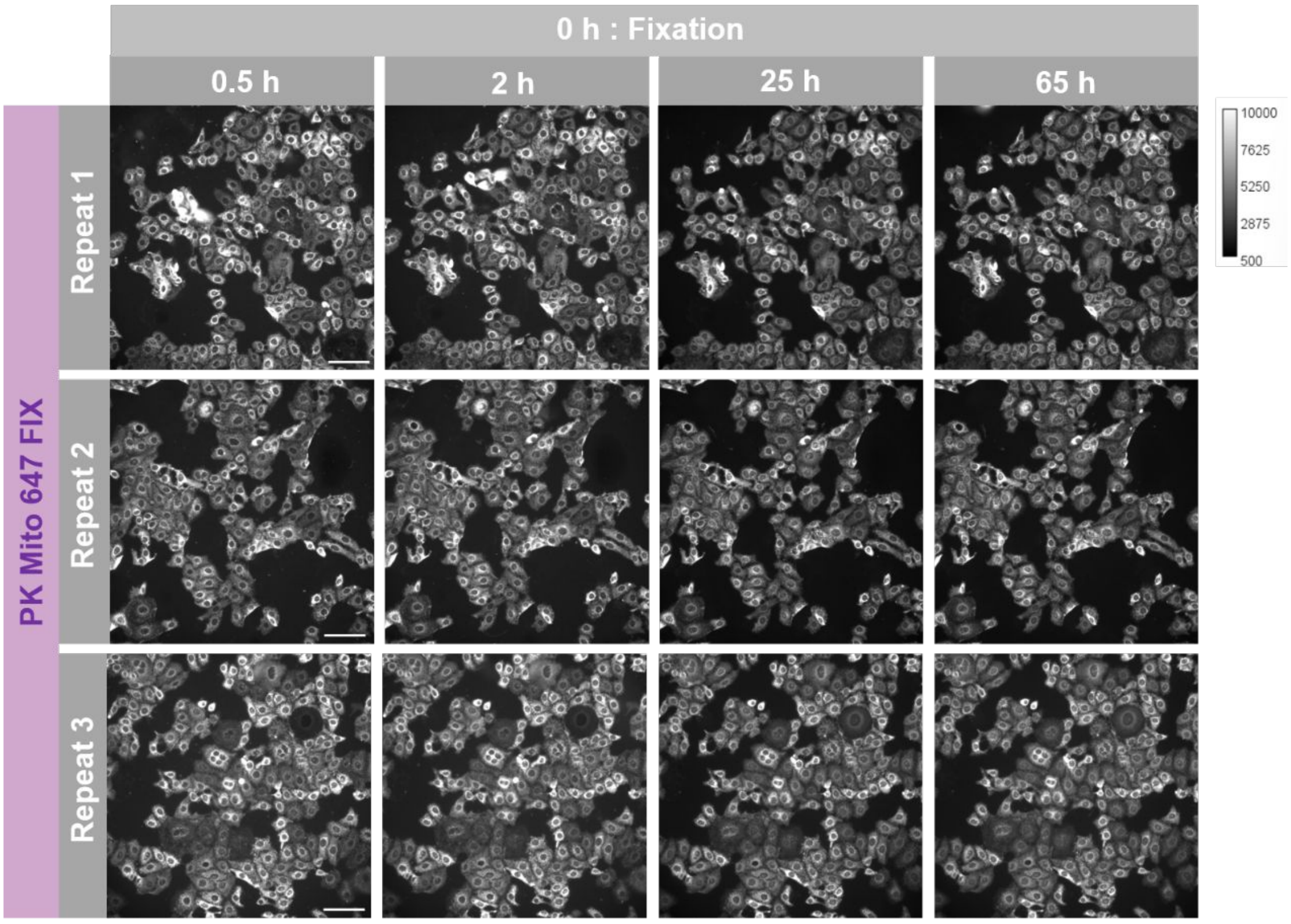

Fig. S12 Long-term fluorescence retention of PK Mito 647 FIX after fixation under high-content imaging.

HeLa cells were stained with 400 nM PK Mito 647 FIX for 10 min and then fixed with 2.5% glutaraldehyde (GA) at t=0 h. Representative fields from three independent experiments are shown, with one field per time point per experiment acquired on the Operetta high-content system at 0.5 h, 2 h, 25 h, and 65 h after fixation. For each experiment, seven fields were imaged, and the panels display a representative field captured and displayed with identical acquisition and display settings across time to enable direct comparison of fluorescence retention. Scale bars, 100 µm.

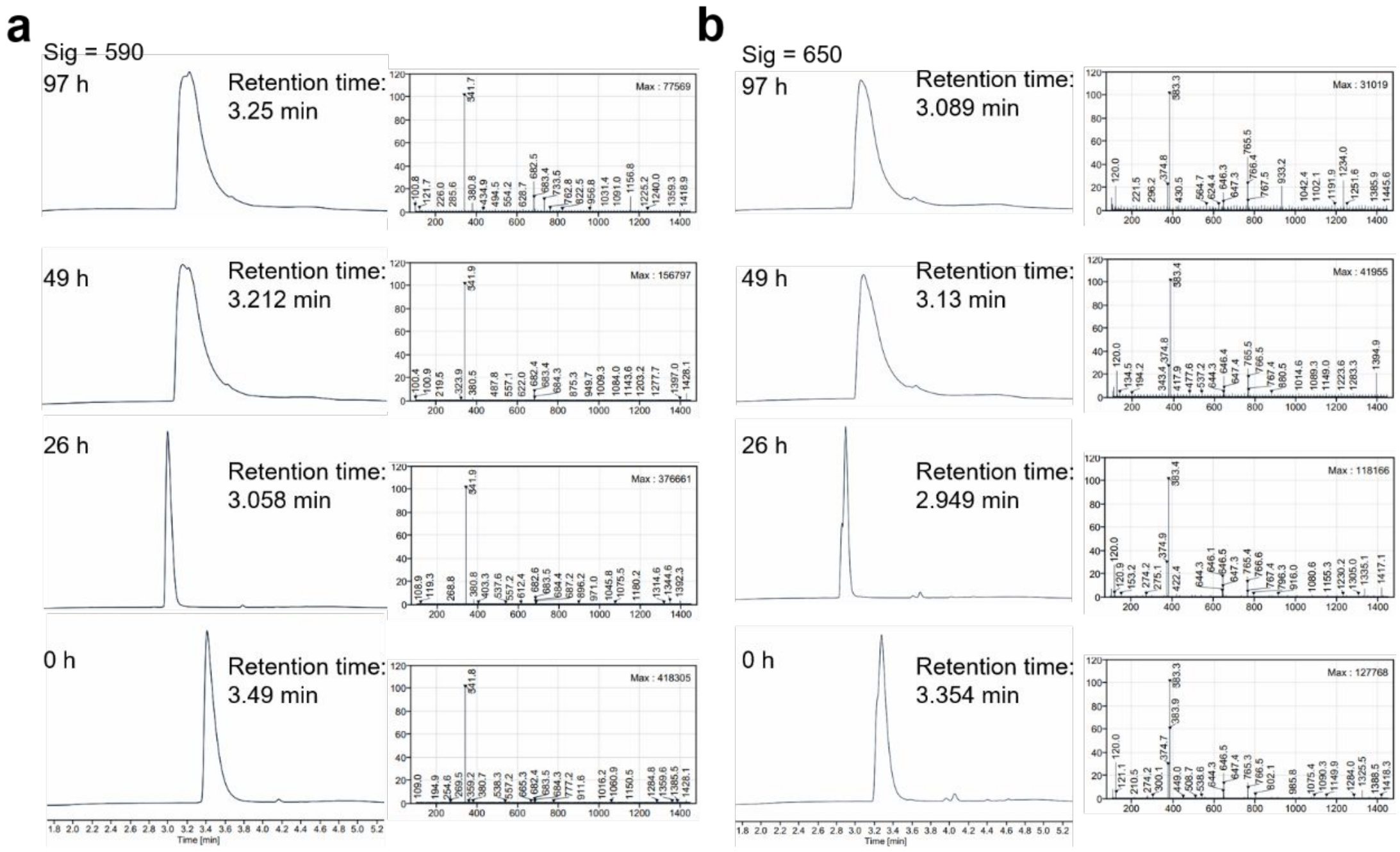

Fig. S13 Stability of PK Mito 590 FIX and PK Mito 647 FIX in water assessed by HPLC–MS.

a. PK Mito 590 FIX was prepared at 150 µM in water and stored at room temperature. Aliquots were analyzed by HPLC–MS at 0 h, 26 h, 49 h, and 97 h. The chromatograms (Sig = 590) show a single dominant peak at each time point with the indicated retention time, and the corresponding mass spectra confirm the expected molecular ion, indicating good stability in aqueous solution over the tested period.

b. The same procedure was applied to PK Mito 647 FIX (150 µM in water, room temperature), with analyses at 0 h, 26 h, 49 h, and 97 h. A single dominant chromatographic peak (Sig = 647) and consistent mass spectra were observed.

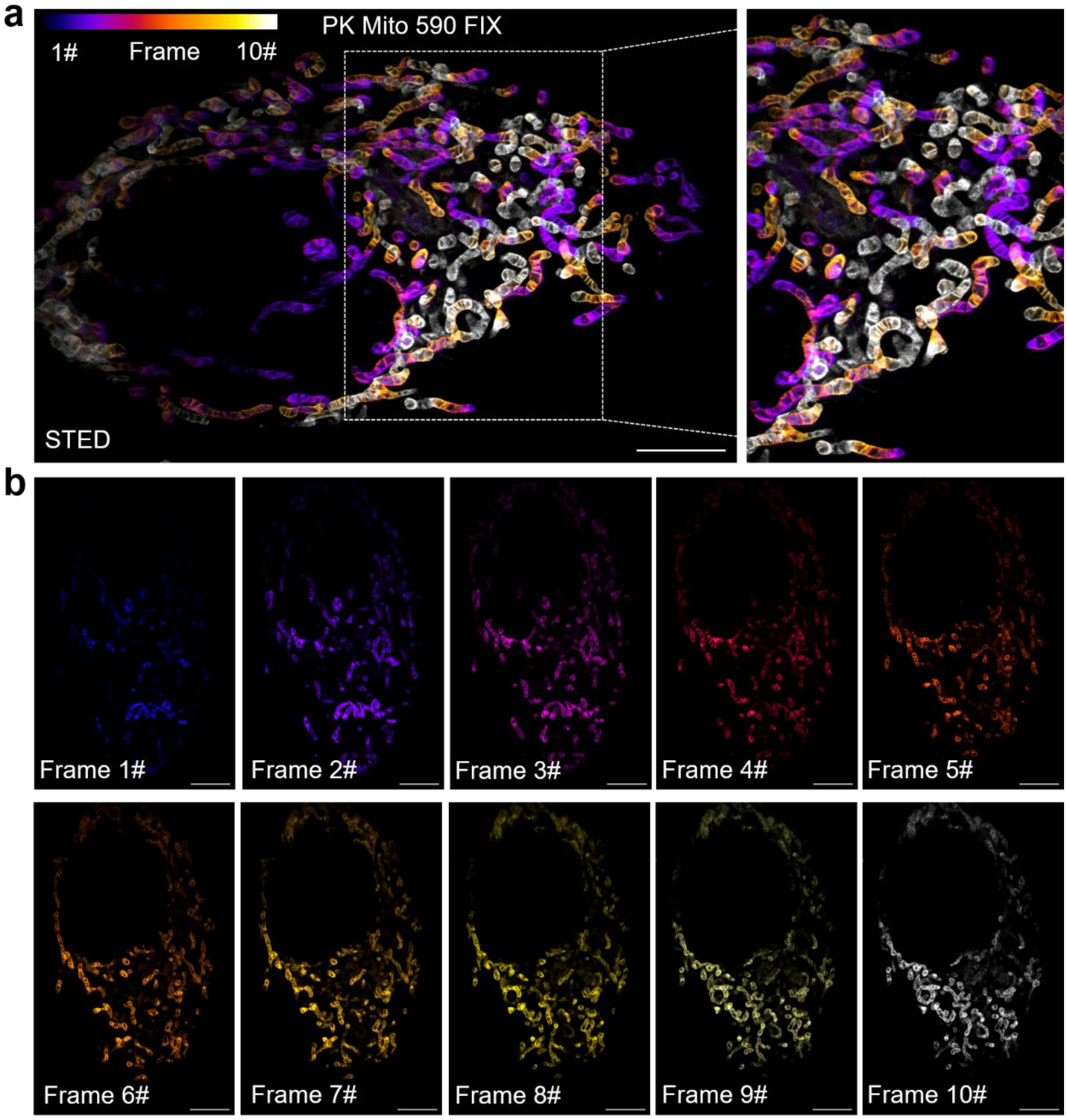

Fig. S14 Visualization of mitochondrial networks using PK Mito 590 FIX in multi-plane STED imaging.

a. Maximum intensity depth-coded projection of mitochondria labeled with PK Mito 590 FIX in a representative cell. The color spectrum indicates z-position within a 2 μm range (−1.0 to +1.0 μm, Frame 1# to Frame 10#). An enlarged view of the selected region is shown on the right. Scale bar, 5 μm.

b. Individual optical sections (Frames 1-10) acquired by STED microscopy at 0.2 μm z-intervals, corresponding to the color-coded depths shown in (a). Scale bars, 5 μm.

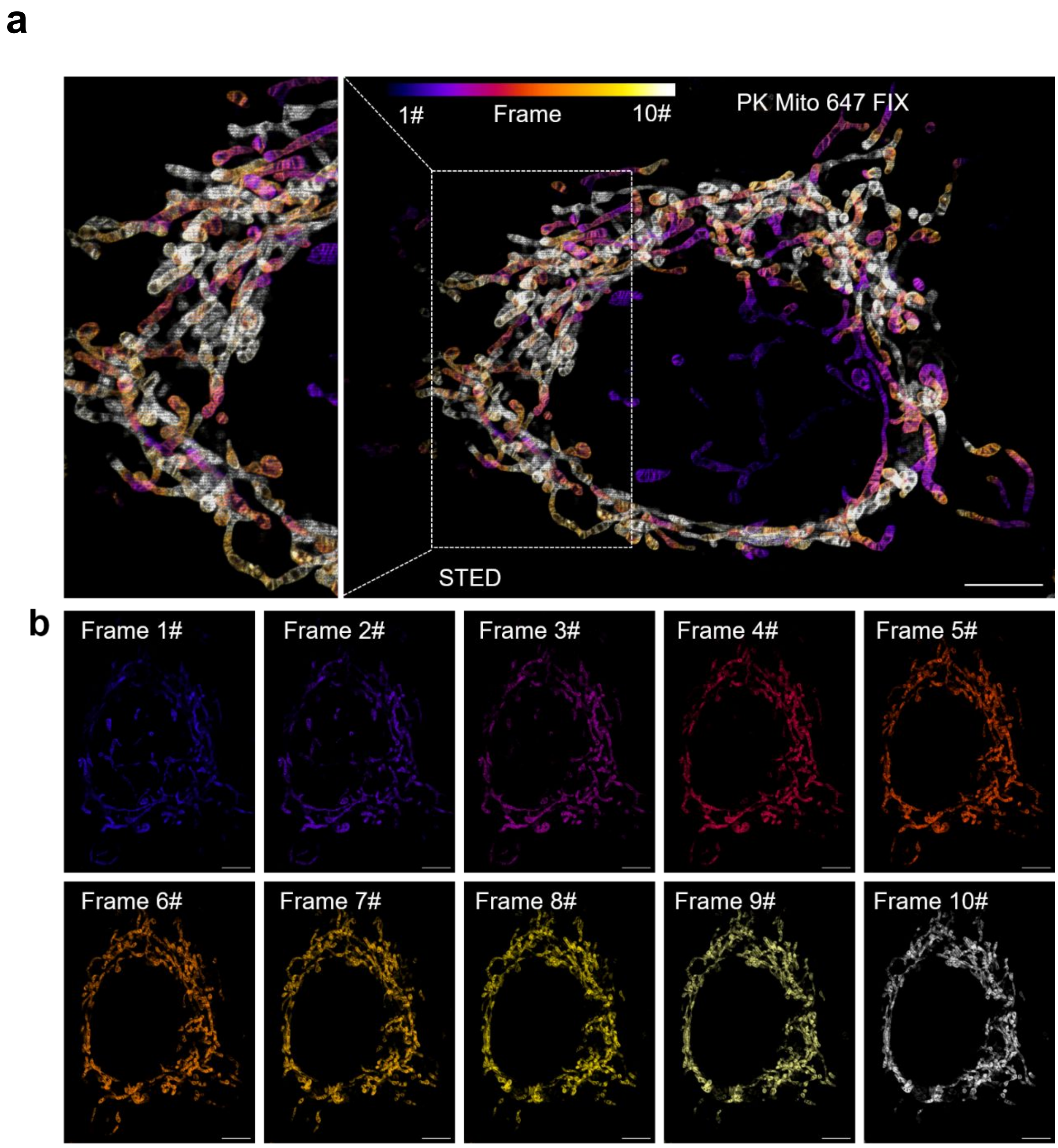

Fig. S15 Visualization of mitochondrial networks using PK Mito 647 FIX in multi-plane STED imaging.

a. Maximum intensity depth-coded projection of mitochondria labeled with PK Mito 647 FIX in a representative cell. The color spectrum indicates z-position within a 2 µm range (−1.0 to +1.0 µm, Frame 1# to Frame 10#). An enlarged view of the selected region is shown on the right. Scale bar, 5 µm.

b. Individual optical sections (Frames 1-10) acquired by STED microscopy at 0.2 µm z-intervals, corresponding to the color-coded depths shown in (a). Scale bars, 5 µm.

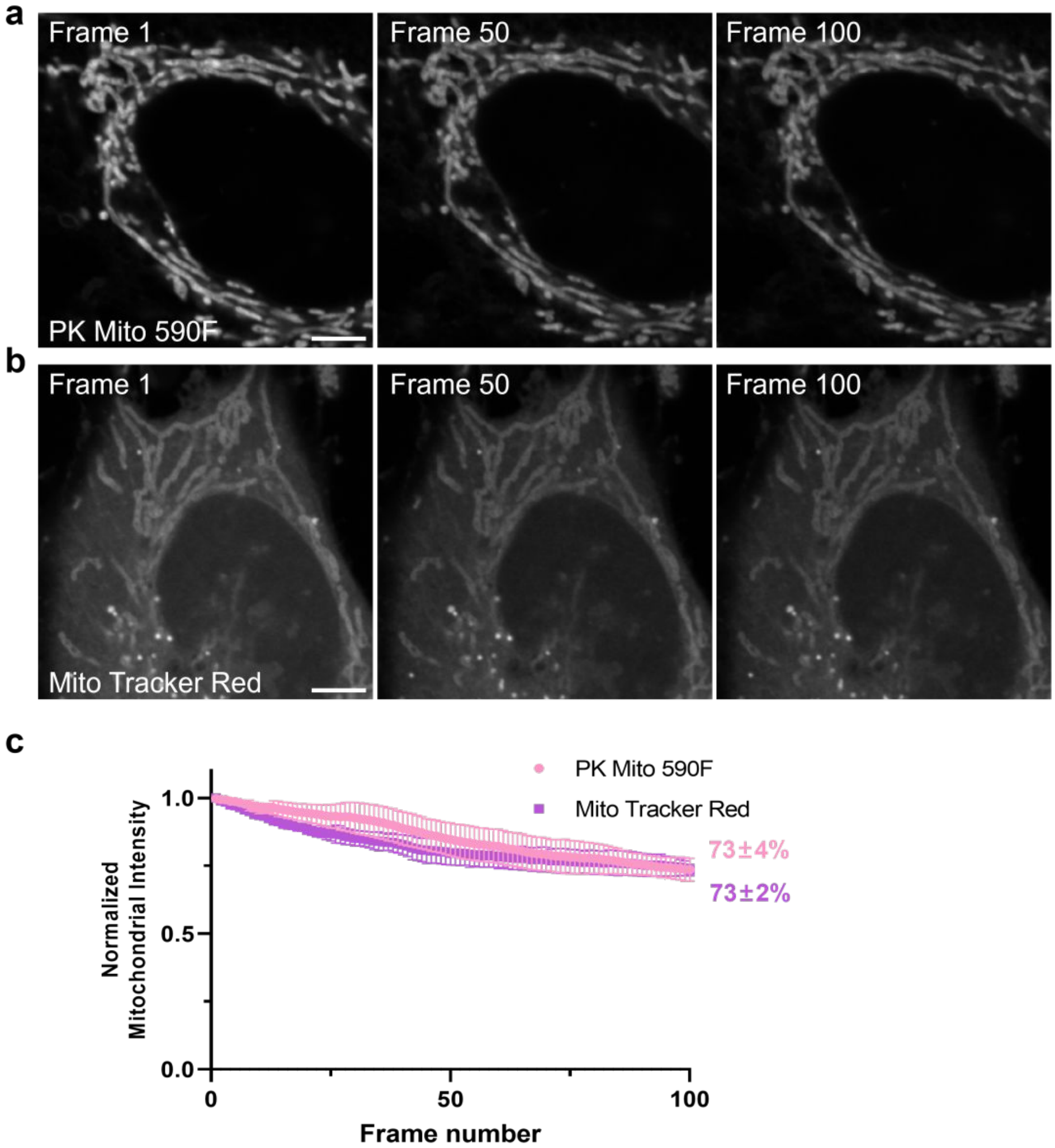

Fig. S16 Photostability of PK Mito 590 FIX and MitoTracker Red under continuous confocal scanning.

a. Confocal images of PK Mito 590 FIX-stained, GA-fixed HeLa cells at frames 1, 50, and 100.

b. Confocal images of MitoTracker Red-stained, GA-fixed HeLa cells at frames 1, 50, and 100.

c. Normalized mitochondrial fluorescence decay curves. PK Mito 590 FIX and MitoTracker Red each retained approximately 73% of the initial fluorescence after 100 scans. Frame size, 30 × 30 µm. Scale bars, 5 µm.

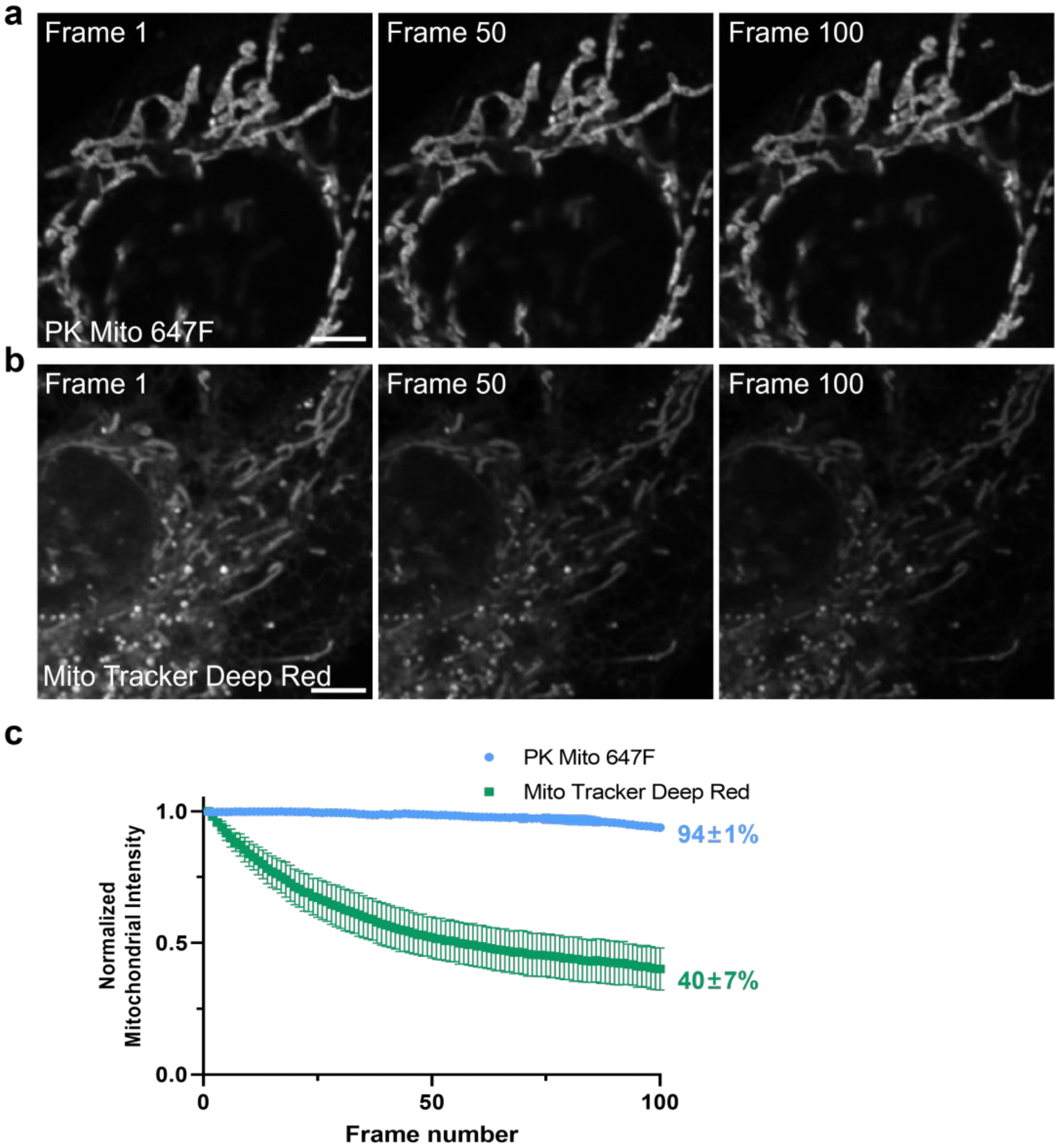

Fig. S17 Photostability of PK Mito 647 FIX and MitoTracker Deep Red under continuous confocal scanning.

a. Confocal images of PK Mito 647 FIX-stained, GA-fixed HeLa cells at frames 1, 50, and 100.

b. Confocal images of MitoTracker Deep Red-stained, GA-fixed HeLa cells at frames 1, 50, and 100.

c. Normalized mitochondrial fluorescence decay curves. PK Mito 647 FIX retained approximately 94% of the initial fluorescence after 100 scans, whereas MitoTracker Deep Red retained approximately 40%. Frame size, 30 × 30 µm. Scale bars, 5 µm.

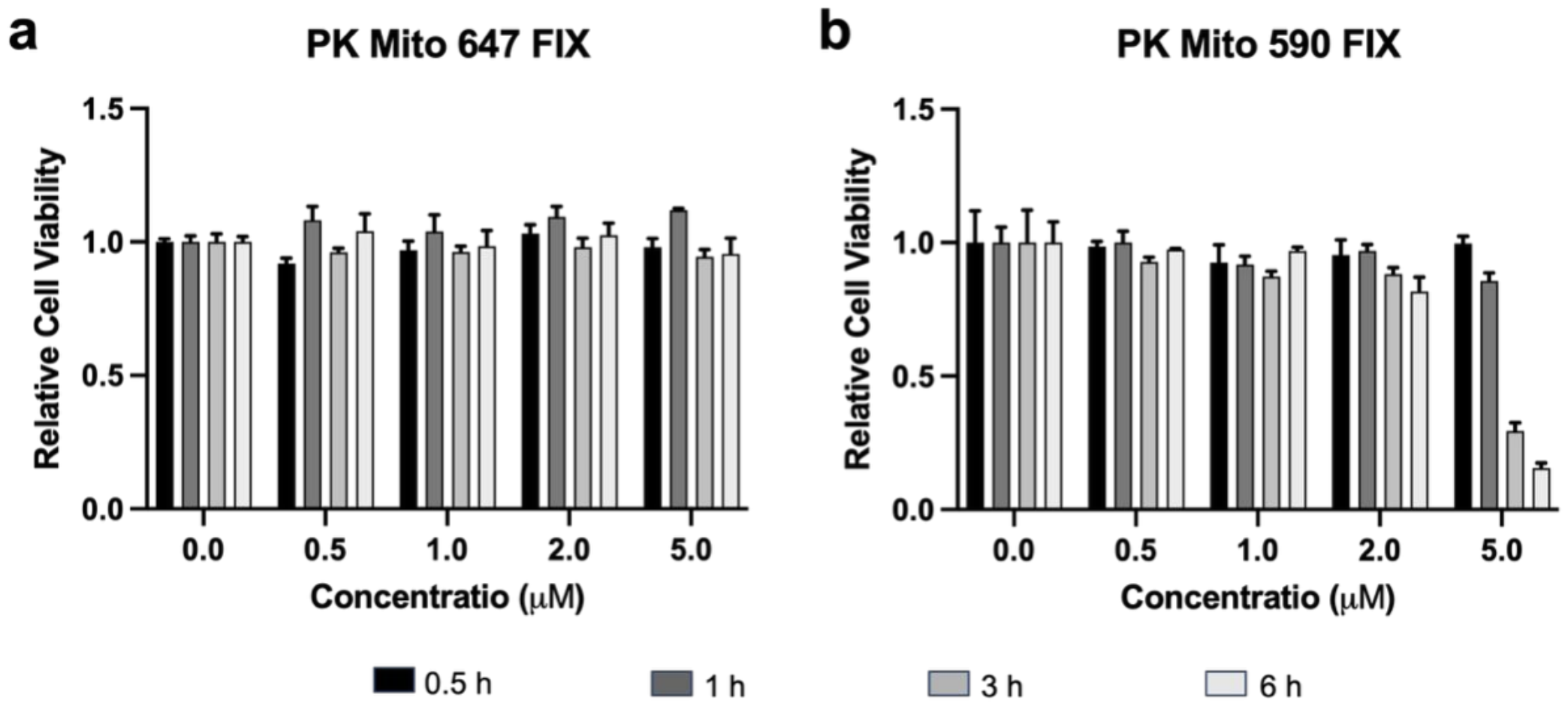

Fig. S18 CCK-8 cell viability analysis of PK Mito FIX dyes.

a. HeLa cells were incubated with PK Mito 647 FIX at the indicated concentrations and time points.

b. HeLa cells were incubated with PK Mito 590 FIX at the indicated concentrations and time points. Cell viability was normalized to the untreated control. Data are shown as mean ± SEM.

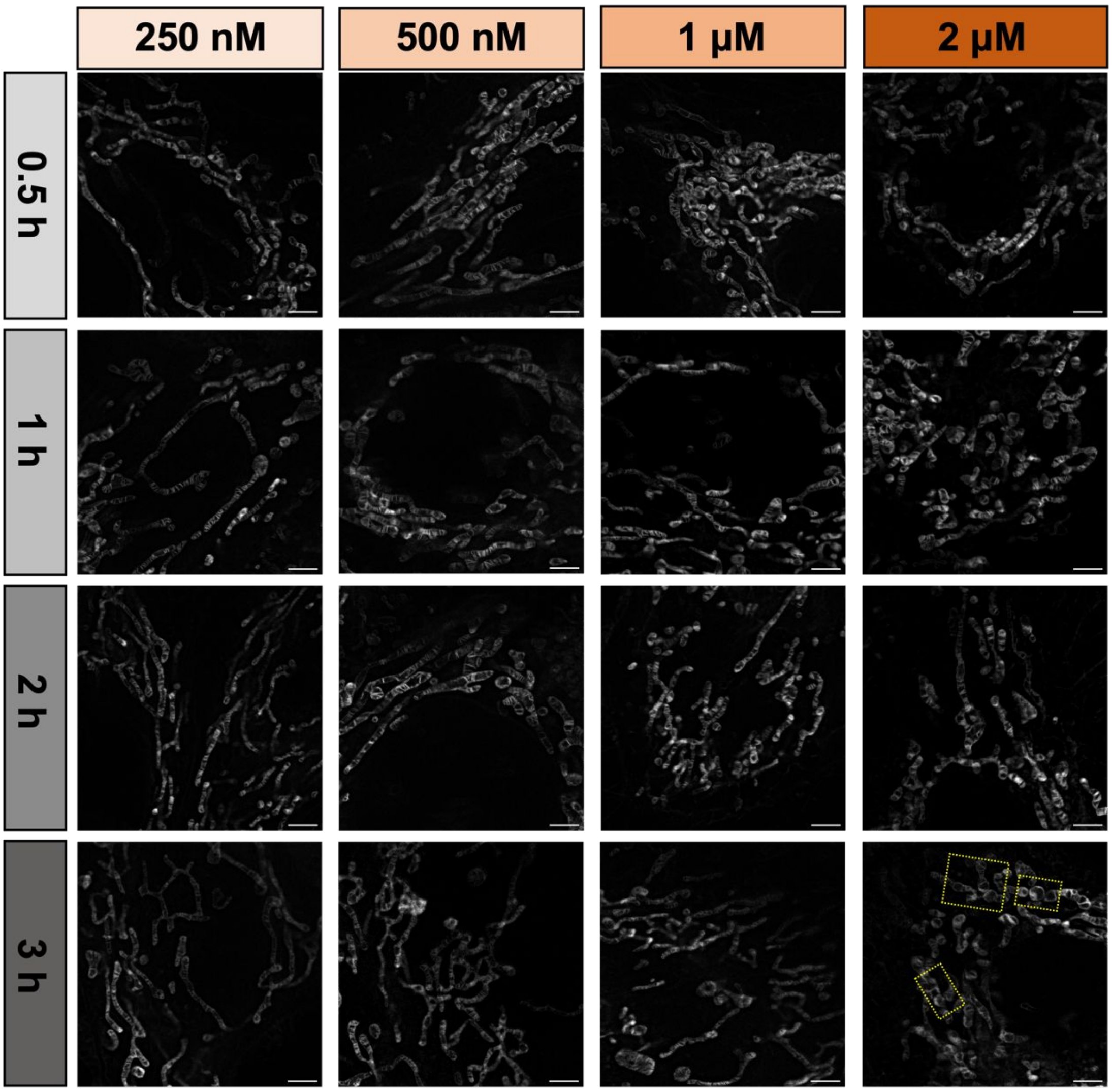

Fig. S19 Concentration-and time-dependent effects of PK Mito 590 FIX staining on mitochondrial morphology.

HeLa cells were incubated with 0.25, 0.5, 1, or 2 μM PK Mito 590 FIX for 0.5, 1, 2 or 3 h and subsequently examined by STED microscopy. Yellow dashed boxes indicate representative regions with evident mitochondrial rounding or swelling. Scale bars, 2 μm.

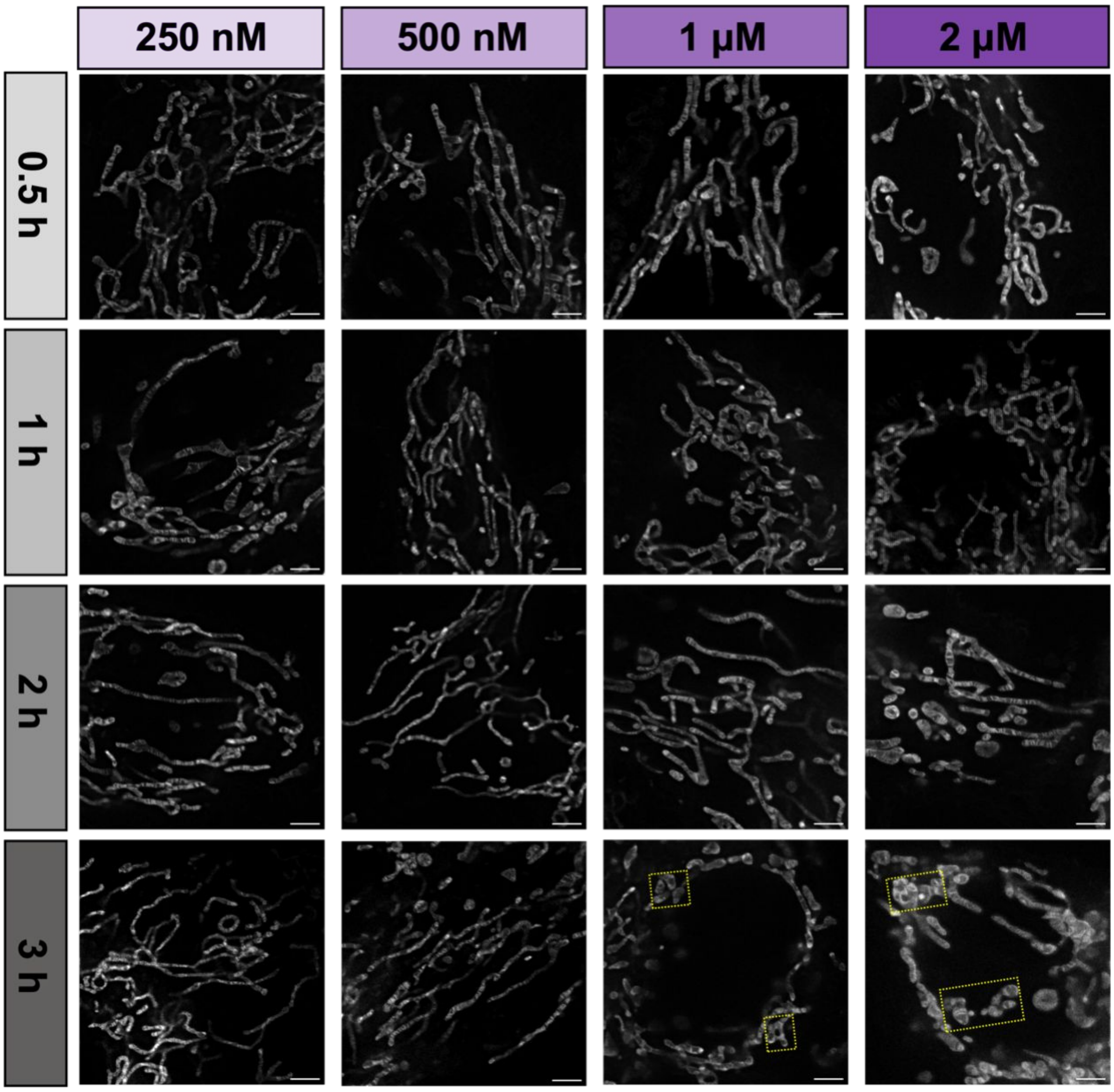

Fig. S20 Concentration- and time-dependent effects of PK Mito 647 FIX staining on mitochondrial morphology.

HeLa cells were incubated with 0.25, 0.5, 1, or 2 μM PK Mito 647 FIX for 0.5, 1, 2, or 3 h and subsequently examined by STED microscopy. Yellow dashed boxes indicate representative regions with evident mitochondrial rounding or swelling. Scale bars, 2 μm.

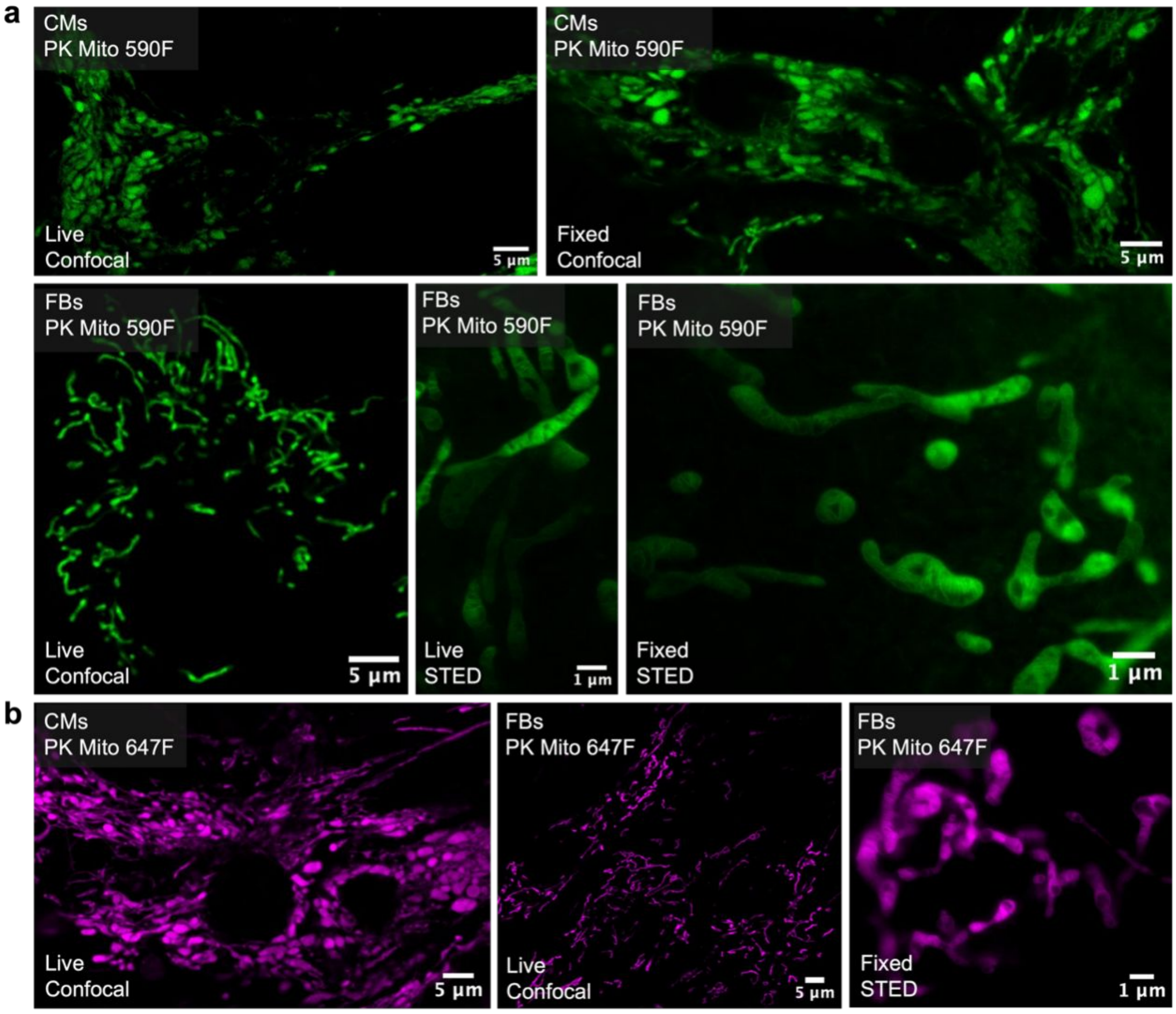

Fig. S21 Validation of PK Mito FIX dyes in neonatal cardiomyocytes and fibroblasts.

a,b: Neonatal cardiomyocytes (CMs) and fibroblasts (FBs) were stained with PK Mito 590 FIX (a) or PK Mito 647 FIX (b), followed by live-cell imaging or fixation with GA and post-fixation imaging, as indicated. Scale bars are indicated in each image.

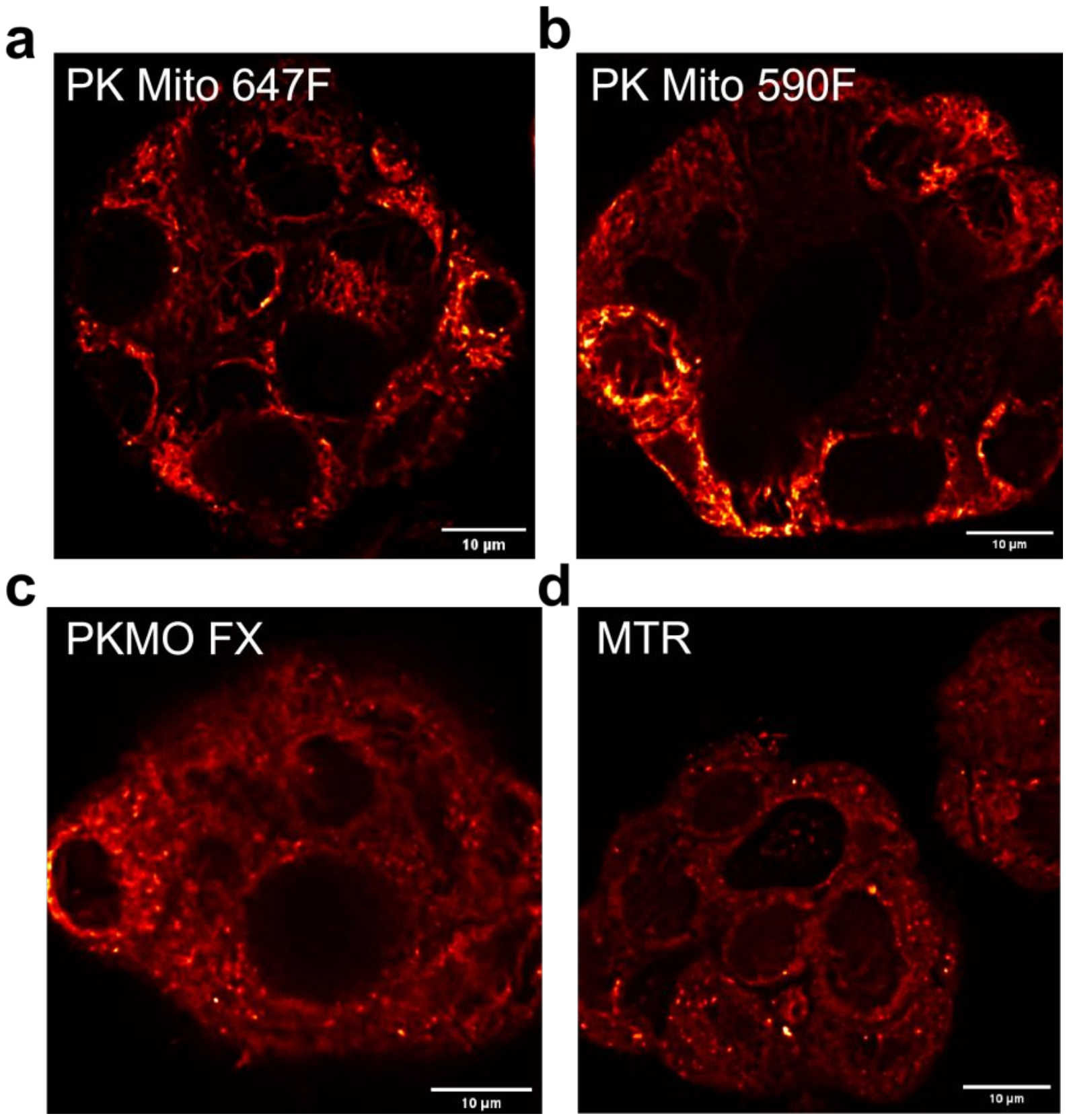

Fig. S22 Confocal images of patient-derived tumor-like cell clusters (PTCs) labeled with four different fixable mitochondrial probes.

Representative confocal images of PTCs labeled with 500 nM PK Mito 647 FIX (a), PK Mito 590 FIX (b), PKMO FX (c), or MitoTracker Red (MTR) (d) for 1.5 h and subsequently fixed with 2.5% GA. Scale bars, 10 μm.

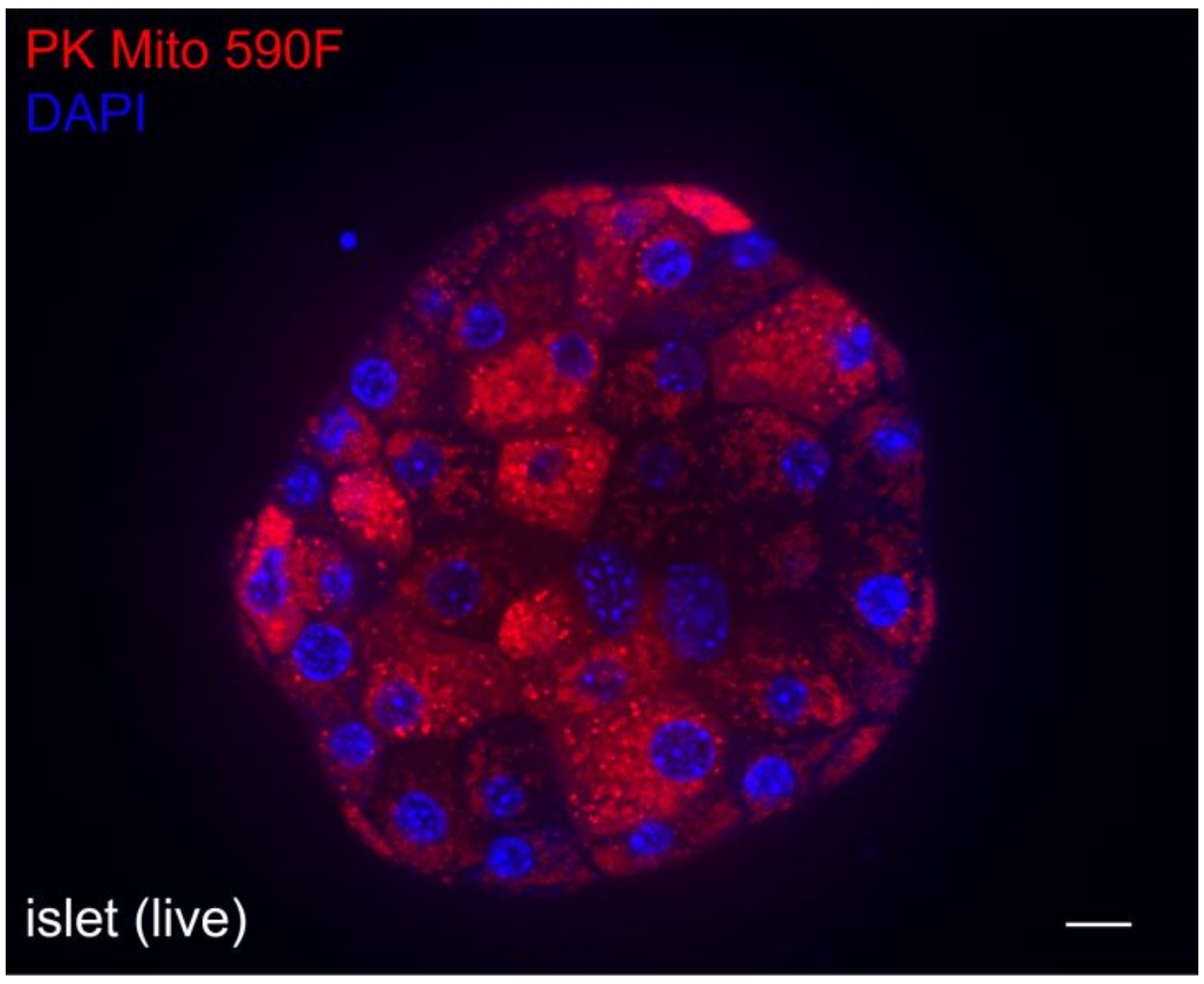

Fig. S23 Confocal image of mitochondria in the live isolated mouse islet tissue.

Representative confocal images of live isolated mouse islet labeled with PK Mito 590 FIX (mitochondria, red) and Hochest (nucleus, blue). Scale bars, 10 μm.

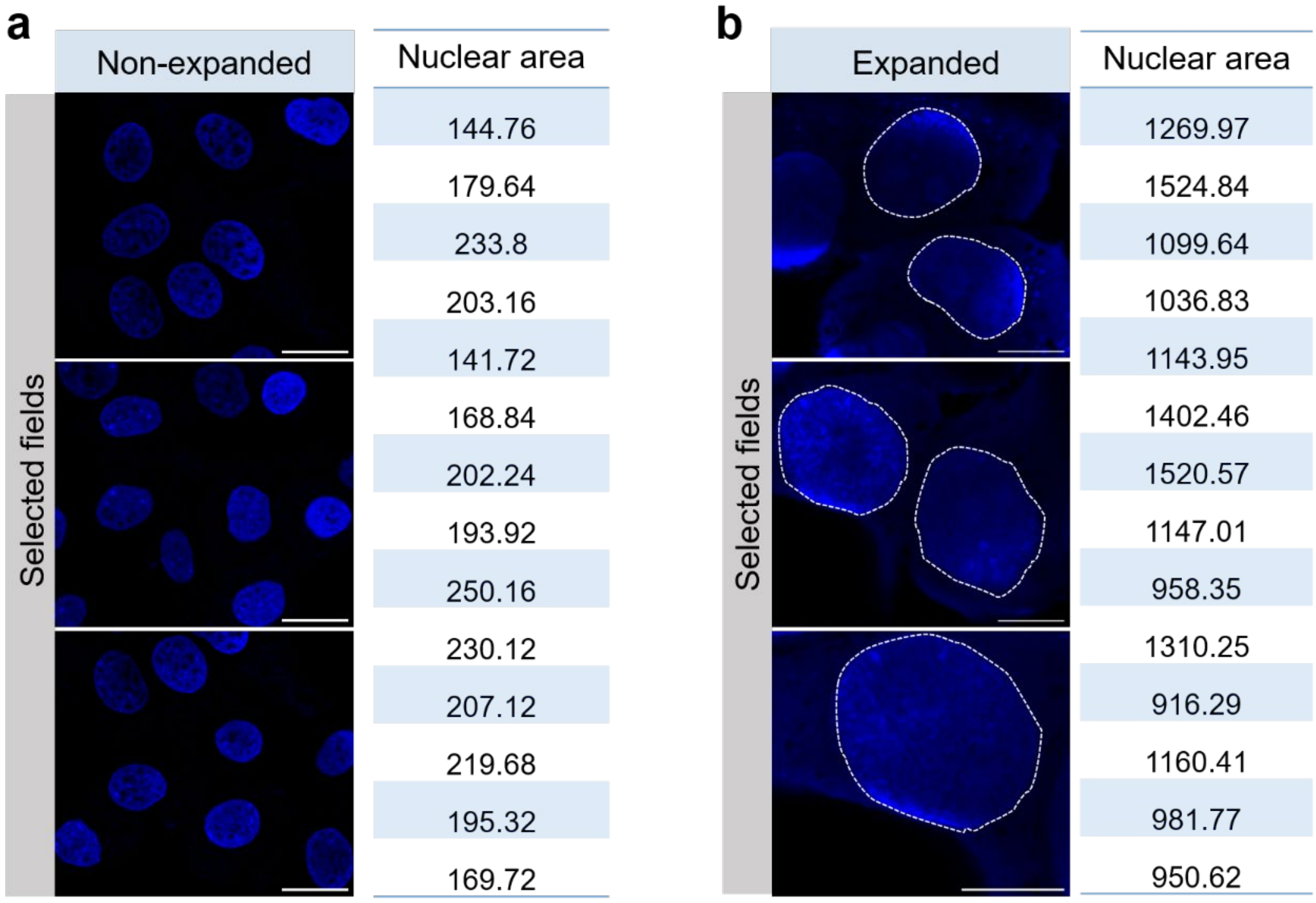

Fig. S24 Nuclear area measurements in non-expanded and expanded samples.

a. Non-expanded cells stained with SYBR Green and their corresponding nuclear area. Scale bars, 20 µm.

b. Expanded cells stained with SYBR Green (outlined by white dashed lines) and their nuclear area. All the areas were measured using ImageJ. Scale bars, 20 µm.

Chemical synthesis and characterization

General experimental information

Unless otherwise mentioned, all the chemicals were purchased at the highest commercial quality and used without further purification unless otherwise stated. Anhydrous dichloromethane (DCM), N,N-dimethylformamide (DMF), acetonitrileand (ACN), tetrahydrofuran (THF) were purchased from Innochem (China). All reactions were carried out under a nitrogen atmosphere with dry solvents unless otherwise mentioned.

Reactions were monitored by Thin Layer Chromatography on plates (GF254) (Yucheng Chemicals) using UV light as visualizing agent or by LC/MS (2.1 mm × 50 mm 1.7 μm C18 column; 2 μL injection; 5-100% CH_3CN/H_2O, linear gradient, with constant 0.1 % v/v formic acid additive; 6-10 min run; 0.5 mL/min flow; ESI; positive or negative ion mode; UV detection with ACQUITY PDA). If not specially mentioned, flash column chromatography uses silica gel (200-300 mesh, Bidepharm). Preparative HPLC separations were performed using Teledyne Isco EZ Prep UV-Vis and a RediSep Prep C18 column (100 Å, 5 μm, 20 × 150 mm).

NMR spectra were recorded on Brüker Advance 400 (^{1}H 400 MHz) and are calibrated using residual undeuterated solvent (CD_3OD at 3.31 ppm ^{1}H NMR). Data for ^{1}H NMR spectra are reported as follows: chemical shift (δ ppm), multiplicity (s = singlet, d = doublet, t = triplet, q = quartet, dd = doublet of doublets, dt = triplet of doublets, m = multiplet, br = broad), coupling constant (Hz), integration. High-resolution mass spectrometric data were obtained using Waters Acquity I class UPLC synapt G2-SI using ESI (electrospray ionization).

Scheme 1. Synthesis of ester-bond fixable mitochondrial dye candidates.

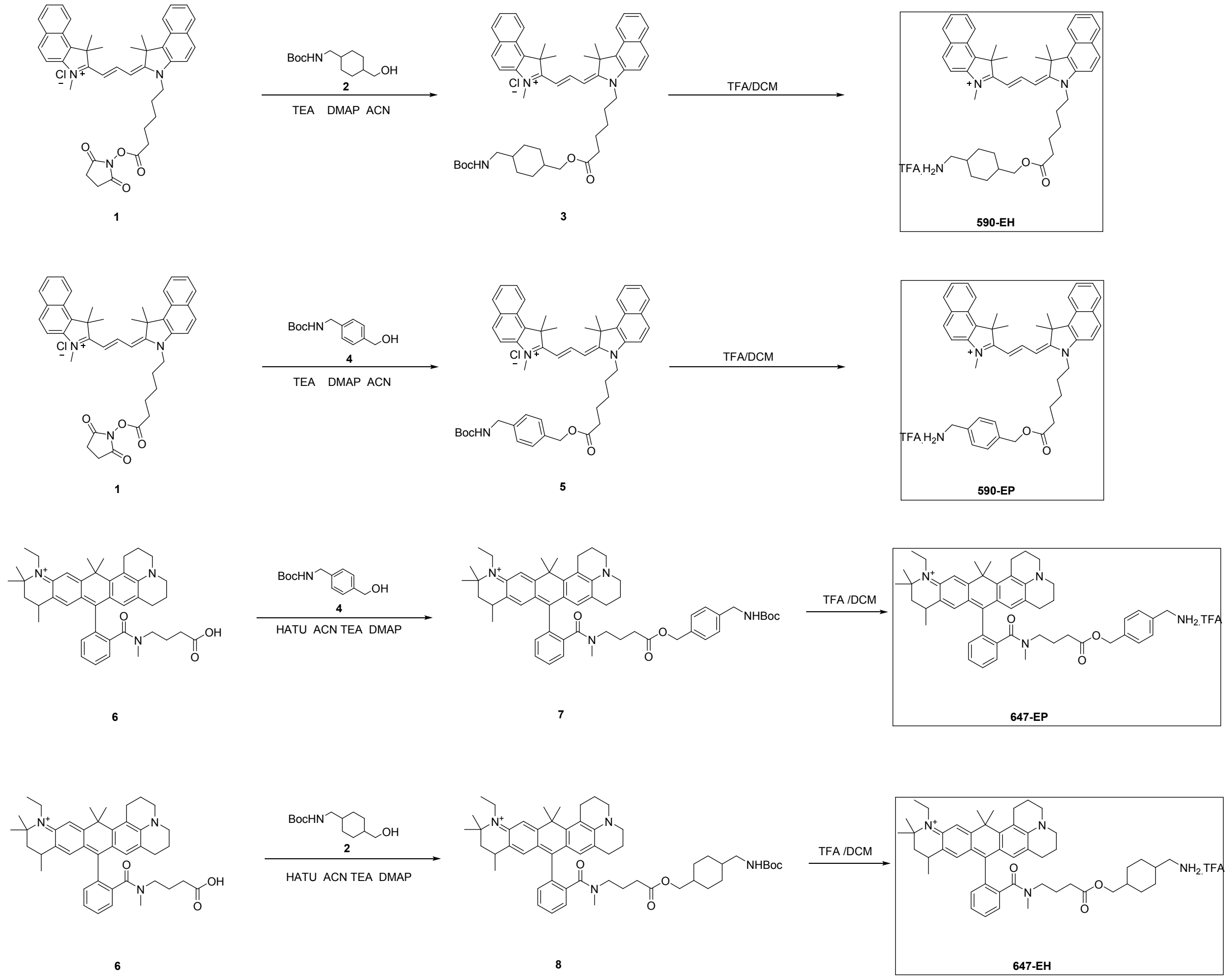

Synthesis of compound 3

To a stirred mixture of compound **1** (10.0 mg, 14.5 μmol, CONFLUORE, China), 4-dimethylaminopyridine (DMAP, 0.354 mg, 2.90 μmol), and triethylamine (TEA, 4.40 mg, 43.5 μmol) in acetonitrile (ACN, 1.0 mL) was added compound **2** (35.3 mg, 145 μmol) at room temperature. The mixture was then heated at 40°C and stirred for 24 h. Then the mixture was concentrated under reduced pressure to yield a residue, which was purified by reversed-HPLC (ACN/0.05% HCl in water, mobile phase in a gradient of 5-60% acetonitrile) to afford compound **3** (6.20 mg, 7.57 μmol, 52%) as a purple solid.

ESI-MS: m/z calculated for $C_{51}H_{64}N_3O_4^+$: $[M]^+$ 782.49; found: 782.45.

^{1}H NMR (400 MHz, Methanol-d_4) δ 8.81-8.76 (m, 1H), 8.30 (d, *J* = 8.0 Hz, 2H), 8.09-8.02 (m, 4H), 7.70-7.63 (m, 4H), 7.55-7.51 (m, 2H), 6.51 (d, *J* = 16.0 Hz, 2H), 4.30 (t, *J* = 8.0 Hz, 2H), 3.85-3.79 (m, 5H), 2.82 (d, *J* = 8.0 Hz, 2H), 2.39-2.37 (m,

2H), 2.10 (s, 12H), 1.94-1.92 (m, 2H), 1.75-1.68 (m, 7H), 1.60-1.53 (m, 4H), 1.42 (s, 8H), 0.90-0.84 (m, 4H).

Synthesis of compound 590-EH

To a stirred mixture of compound **3** (6.20 mg, 7.57 μmol) dissolved in dichloromethane (DCM, 0.8 mL) was added trifluoroacetic acid (TFA, 0.2 mL) at room temperature. The reaction mixture was then stirred at the same temperature for 2 h. The mixture was then concentrated under reduced pressure to yield a crude residue, which was purified by reversed-HPLC (ACN/0.05% TFA in water, mobile phase in a gradient of 5−50% acetonitrile) to afford the compound **590-EH** (5.02 mg, 85%) as a purple solid.

ESI-MS: m/z calculated for $C_{51}H_{64}N_3O_4^+$: $[M]^+$ 682.44; found 682.44.

^{1}H NMR (400 MHz, Methanol-d_4) δ 8.78-8.75 (t, J = 12.0 Hz, 1H), 8.30 (d, J = 8.0 Hz, 2H), 8.08- 8.02 (m, 4H), 7.70 - 7.63 (m, 4H), 7.55-7.53 (m, 2H), 6.49 (dd, J = 12.0, 4.0 Hz, 2H), 4.29 (t, J = 8.0 Hz, 2H), 3.85-3.82 (m, 5H), 2.74 (d, J = 8.0 Hz, 2H), 2.38 (t, J = 8.0 Hz, 2H), 2.11 (s, 12H), 1.96-1.92 (m, 2H), 1.80-1.74 (m, 6H), 1.57-1.53 (m, 4H), 1.01-0.95 (m, 4H).

Synthesis of compound 5

To a stirred mixture of compound **1** (1.30 mg, 1.88 μmol), DMAP (0.046 mg, 0.377 μmol) and TEA (0.572 mg, 5.64 μmol) in ACN (0.3 mL) was added compound **4** (8.92 mg, 37.6 μmol) at room temperature. The mixture was then heated at 40°C and stirred for 36 h. Then the mixture was concentrated under reduced pressure to afford a residue, which was purified by reversed-HPLC (ACN/0.05% HCl in water, mobile phase in a gradient of 5-60% acetonitrile) to afford compound **5** (0.40 mg, 0.49 μmol, 26%) as a purple solid.

ESI-MS: m/z calculated for $C_{51}H_{58}N_3O_4^+$ $[M]^+$ 776.44; found 776.68.

Synthesis of compound 590-EP

To a stirred mixture of compound **5** (0.40 mg, 0.49 μmol) in DCM (0.2 mL) was

added TFA (0.1 mL) at room temperature. The mixture was then stirred at this temperature for 2 h. Then the mixture was concentrated under reduced pressure to afford a residue, which was purified by reversed-HPLC (ACN/0.05% TFA in water, mobile phase in a gradient of 3−50% acetonitrile) to afford the compound **590-EP** (0.304 mg, 80%) as a purple solid.

ESI-MS: m/z calculated for $C_{46}H_{50}N_3O_2^+$ $[M]^+$ 676.39; found 676.52.

Synthesis of compound 7

To a stirred mixture of compound **6** (4.0 mg, 6.18 μmol, AAT Bioquest), DMAP (0.151 mg, 1.23 μmol) and TEA (1.88 mg, 18.6 μmol) in ACN (0.6 mL) were added HATU (5.06 mg, 13.3 μmol) and compound **4** (14.7 mg, 61.8 μmol) at room temperature. After addition, the mixture was heated at 45°C and stirred for 24 h. Then the mixture was concentrated under reduced pressure to afford a residue, which was purified by reversed-HPLC (ACN/0.05% HCl in water, mobile phase in a gradient of 5-60% acetonitrile) to afford compound **7** (4.50 mg, 5.19 μmol, 84%) as a blue solid.

ESI-MS: m/z calculated for $C_{55}H_{69}N_4O_5^+$ $[M]^+$ 865.53; found 865.59.

^{1}H NMR (400 MHz, Methanol-*d*$_4$) δ 7.62-7.59 (m, 2H), 7.52 -7.49 (m, 1H), 7.35-7.31 (m, 1H), 7.26 (s, 4H), 6.83(s, 1H), 6.75-6.72 (m, 2H), 5.84 (s, 1H), 5.03- 4.96 (m, 2H), 4.20 (s, 2H), 3.77-3.69 (m, 1H), 3.64-3.56 (m, 1H), 3.51-3.25 (m, 5H), 3.10-3.01 (m, 3H), 2.89-2.78 (m, 3H), 2.74-2.65 (m, 1H), 2.51-2.48 (m, 3H), 1.84 -1.80 (m, 7H), 1.75-1.71 (m, 3H), 1.47-1.40 (m, 15H), 1.30-1.27 (m, 8H), 0.98-0.94 (m, 3H).

Synthesis of compound 647-EP

To a mixture of compound **7** (4.50 mg, 5.19 μmol) in DCM (0.4 mL) was added TFA (0.1 mL) at room temperature. After addition, the mixture was stirred at this temperature for 1.5 h. Then the mixture was concentrated under reduced pressure to afford a residue, which was purified by reversed-HPLC (ACN/0.05% TFA in water, mobile phase in a gradient of 3-50% acetonitrile) to afford the compound **647-EP** (3.10 mg, 69%) as a blue solid.

ESI-MS: m/z calculated for $C_{50}H_{61}N_4O_3^+$ $[M]^+$ 765.47; found 765.50.

^{1}H NMR (400 MHz, Methanol-d_4) δ 7.72-7.70 (m, 2H), 7.62-7.58 (m, 1H), 7.48-7.41 (m, 5H), 6.93(s, 1H), 6.85-6.80 (m, 2H), 5.12 (s, 2H), 4.20 (s, 2H), 4.14 (s, 2H), 3.84-3.80 (m, 1H), 3.70-3.66 (m, 1H), 3.59-3.54 (m, 4H), 3.40- 3.35 (m, 1H), 3.22-3.15 (m, 3H), 2.97-2.87 (m, 3H), 2.80-2.76 (m, 1H), 2.68-2.60 (m, 3H), 2.05-2.04 (m, 3H), 1.96-1.90 (m, 6H), 1.84-1.83 (m, 3H), 1.56-1.43 (m, 6H), 1.38-1.34 (m, 6H), 1.06-1.02 (m, 3H).

Synthesis of compound 8

To a mixture of compound **6** (1.75 mg, 2.71 μmol), DMAP (0.066 mg, 0.541 μmol) and TEA (0.822 mg, 8.13 μmol) in ACN (0.4 mL) were added HATU (2.06 mg, 5.42 μmol) and compound **4** (6.59 mg, 27.1 μmol) in turn at room temperature. After addition, the mixture was heated at 50°C and stirred for 24 h. Then the mixture was concentrated under reduced pressure to afford a residue, which was purified by reversed-HPLC (ACN/0.05% HCl in water, mobile phase in a gradient of 5-60% acetonitrile) to afford compound **8** (0.8 mg, 0.92 μmol, 34%) as a blue solid.

ESI-MS: m/z calculated for $C_{55}H_{75}N_4O_5^+$ $[M]^+$ 871.57; found 871.44.

Synthesis of compound 647-EH

To a mixture of compound **8** (0.8 mg, 0.92 μmol) in DCM (0.3 mL) was added TFA (0.1 mL) at room temperature. After addition, the mixture was stirred at this temperature for 1.5 h. Then the mixture was concentrated under reduced pressure to afford a residue, which was purified by reversed-HPLC (ACN/0.05% TFA in water, mobile phase in a gradient of 3−50% acetonitrile) to afford the compound **647-EH** (0.66 mg, 83%) as a blue solid.

ESI-MS: m/z calculated for $C_{50}H_{67}N_4O_3^+$ $[M]^+$ 771.52; found 771.43.

References:

1. Peng, X.; Ren, H.; Yang, L.; Tong, S.; Zhou, R.; Long, H.; Wu, Y.; Wang, L.; Wu, Y.; Zhang, Y.; Shen, J.; Zhang, J.; Qiu, G.; Wang, J.; Han, C.; Zhang, Y.; Zhou, M.; Zhao, Y.; Xu, T.; Tang, C.; Chen,

Z.; Liu, H.; Chen, L., Readily releasable β cells with tight Ca^{2+}–exocytosis coupling dictate biphasic glucose-stimulated insulin secretion. *Nature Metabolism* **2024,** *6* (2), 238-253.
